# Detecting and correcting for bias in Mendelian randomization analyses using Gene-by-Environment interactions

**DOI:** 10.1093/ije/dyy204

**Published:** 2018-11-20

**Authors:** Wes Spiller, David Slichter, Jack Bowden, George Davey Smith

**Affiliations:** 1 Population Health Sciences, University of Bristol, Bristol, UK; 2 Department of Economics, Binghamton University, State University of New York, Binghamton, NY, USA

**Keywords:** Mendelian randomization, invalid instruments, pleiotropy, MRGxE, gene–environment interaction

## Abstract

**Background:**

Mendelian randomization (MR) has developed into an established method for strengthening causal inference and estimating causal effects, largely due to the proliferation of genome-wide association studies. However, genetic instruments remain controversial, as horizontal pleiotropic effects can introduce bias into causal estimates. Recent work has highlighted the potential of gene–environment interactions in detecting and correcting for pleiotropic bias in MR analyses.

**Methods:**

We introduce MR using Gene-by-Environment interactions (MRGxE) as a framework capable of identifying and correcting for pleiotropic bias. If an instrument–covariate interaction induces variation in the association between a genetic instrument and exposure, it is possible to identify and correct for pleiotropic effects. The interpretation of MRGxE is similar to conventional summary MR approaches, with a particular advantage of MRGxE being the ability to assess the validity of an individual instrument.

**Results:**

We investigate the effect of adiposity, measured using body mass index (BMI), upon systolic blood pressure (SBP) using data from the UK Biobank and a single weighted allelic score informed by data from the GIANT consortium. We find MRGxE produces findings in agreement with two-sample summary MR approaches. Further, we perform simulations highlighting the utility of the approach even when the MRGxE assumptions are violated.

**Conclusions:**

By utilizing instrument–covariate interactions in MR analyses implemented within a linear-regression framework, it is possible to identify and correct for horizontal pleiotropic bias, provided the average magnitude of pleiotropy is constant across interaction-covariate subgroups.

**Key Messages**

- Instrument–covariate interactions can be used to identify bias due to horizontal pleiotropy in Mendelian randomization (MR) analyses, provided they induce sufficient variation in the association between the genetic instrument and exposure.
- By regressing the gene–outcome association upon the gene-exposure association across interaction-covariate subgroups, MR using Gene-by-Environment interactions (MRGxE) returns estimates of the average pleiotropic effect and the pleiotropy adjusted causal effect.
- The interpretation of MRGxE is analogous to that of MR-Egger regression.
- The approach serves as a test for pleiotropy and can inform instrument selection.

## Introduction

Mendelian randomization (MR) has developed into a multifaceted approach to assessing causal relationships in epidemiology.^1^^,^^2^ In many cases, MR analyses employ genetic variants as instrumental variables (IVs), allowing consistent estimation of causal effects in the presence of unmeasured confounding. This requires candidate variants to be associated with the exposure of interest (IV1), to be independent of confounders of the exposure and outcome (IV2) and to be independent of the outcome outside of the mediating effects of the exposure (IV3).^3^ An instrument satisfying these assumptions is considered valid, although IV2 and IV3 cannot directly tested.

Pleiotropy plays a central role in MR analyses and can be subcategorized into vertical and horizontal forms. Vertical pleiotropy exists in cases where a single genetic variant influences a phenotype, which in turn influences another.^4^ This is the primary mechanism underpinning the utility of MR in causal-effect estimation. However, a particular concern when applying MR is horizontal pleiotropy—occurring when a genetic variant is associated with a study outcome through biological pathways additional to the exposure of interest.^2^^,^^5^ This violates assumption IV3, introducing bias into effect estimates in the direction of the horizontal pleiotropic (henceforth, pleiotropic) effect.^5^^,^^6^ Where multiple instruments are available, one strategy is to combine causal estimates using each individual variant in turn within a meta-analysis framework. Provided the genetic variants are uncorrelated, an inverse-variance-weighted (IVW) estimate will be equivalent to two-stage least-squares (TSLS) regression and, where pleiotropy is suspected, MR-Egger, median and modal regression can be adopted as sensitivity analyses.^4^^,^^5^^,^^7^^,^^8^

In the single instrument setting, Slichter regression has emerged from the econometrics literature as a method for evaluating instrument validity within a potential outcomes framework.^9^^,^^10^ This involves observing or extrapolating to a population subgroup for which the instrument and exposure are independent (defined as a *no-relevance group*) and measuring the corresponding association between the instrument and outcome. The instrument–outcome association for a no-relevance group provides an estimate of pleiotropic effect and allows bias correction within a statistical model. Slichter regression builds upon several key developments in econometrics, in particular the identification and estimation of local average treatment effects put forward by Imbens and Angrist,^10^ and the works of Card,^11^ Conley *et al*.^12^ and Small.^13^

In this paper, we introduce Slichter regression within the context of epidemiology, formalizing the increasing use of gene–environment interactions in assessing instrument validity.^14–20^ We present MR using Gene-by-Environment interactions (MRGxE) as a statistical framework and sensitivity analysis to identify and correct for pleiotropic bias in MR studies using gene–covariate interactions. Importantly, MRGxE can assess the validity of a single instrument, in contrast to methods examining heterogeneity across a set of MR estimates using many instruments, and is not reliant upon the existence of an observed no-relevance group. This represents an improvement upon similar methods such as Pleiotropy Robust Mendelian Randomization (PRMR) that, while sharing a similar intuitive framework, are reliant upon the existence of an actual no-relevance group being observed within the data, severely limiting the applicability of the approach.^21^

Two features differentiate MRGxE from analogous methods in the econometrics literature. First, MRGxE adopts a linear-regression framework as opposed to utilizing local linear regression, improving the ease with which MRGxE can be implemented. Additionally, MRGxE can be applied using both individual- and summary-level data. Such data could be obtained from previously published studies where subgroup-specific estimates are provided or alternatively requested from consortia.

To illustrate the utility of MRGxE, we present an applied example examining the effect of body mass index (BMI) upon systolic blood pressure (SBP), utilizing data from the GIANT consortium and the full release of the UK Biobank (July 2017), respectively.^22^ We find evidence suggesting a positive association between BMI and SBP, and substantial agreement between MRGxE and two-sample summary MR estimates. Finally, we conduct a simulation study demonstrating the effectiveness of the approach under varying conditions.

## Methods

### Non-technical intuition

Consider a situation in which the instrument–exposure association is found to vary between subgroups of the target population. We follow Slichter in defining an observed subgroup for which the instrument does not predict the exposure of interest as a *no-relevance group*.^9^ As a valid IV can only be associated with the outcome of interest through the exposure, it follows that the IV would be independent of the outcome for the no-relevance group. An observed non-zero association for the no-relevance group therefore serves as evidence of pleiotropy.

This intuitive approach has been considered in several epidemiological studies. For example, Chen *et al.*^19^ considered differences in drinking behaviour by gender in East Asian populations within a fixed-effects meta-analysis of the *ALDH2* genetic variant and blood pressure. This interaction has received further attention in work such as Taylor *et al.*^23^ and Cho *et al.*^14^ Previous applications also extend beyond simple gender differences. For example, Tyrrell *et al.* identified genetically predicted BMI as a weaker instrument for participants experiencing lower levels of socio-economic deprivation, utilizing negative controls to examine residual confounding.^16^

In presenting MRGxE, we highlight similarities to the approach of Cho *et al.*,^14^ in which a gender–*ALDH2* interaction term was incorporated within a TSLS model to estimate the effect of alcohol consumption. We clarify how it works when individual-level data are available and crucially demonstrate how MRGxE extends this approach to summary data.

### The MRGxE framework

**Figure 1.**
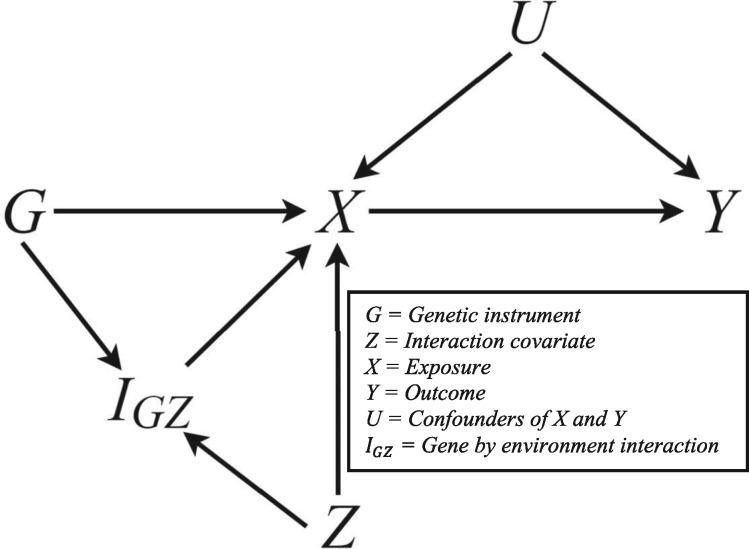
A directed acyclic graph (DAG) showing the assumed relationship between each variable in MRGxE.

Consider an MR study consisting of N participants (indexed by i=1,…,N). For each participant, we record observations of a genetic instrument$\;G_{i}$, an exposure$\;X_{i}$, an outcome$\;Y_{i}$ and a further covariate$\;Z_{i}$, which induces variation in the association between$\;G_{i}$ and$\;X_{i}$ through an interaction $GZ_{i}$. The relationship between each variable is illustrated in Figure 1, with U representing a set of all unmeasured variables confounding X and Y, and $I_{GZ}$ representing the interaction term.

The exposure X is a linear function of G, Z, GZ, U and an independent error term,${\;\epsilon }_{X}$, whilst the outcome Y is a linear function of G, Z,GZ, U,X and an independent error term,$\;\epsilon_{Y}$. Using γ and β to denote regression coefficients for the first- and second-stage models, respectively, a two-stage model can be defined as:

(1) $$X_{i}=\gamma_{0}+\gamma_{1}G_{i}+\gamma_{2}Z_{i}+\gamma_{3}GZ_{i}+U_{i}+\epsilon_{Xi},$$

(2) $$Y_{i}=\beta_{0}+\beta_{1}X_{i}+\beta_{2}G_{i}+\beta_{3}Z_{i}+\beta_{4}GZ_{i}+U_{i}+\epsilon_{Yi}.$$

The causal effect of X on Y is denoted$\;by\;\beta_{1}$ and the pleiotropic effect of the instrument is$\;\beta_{2}$. Note that regressing Y upon X would be prone to confounding bias and applying TSLS would give biased estimates when$\;\beta_{2}\neq 0$. This is demonstrated in the Supplementary Material, available as Supplementary data at *IJE* online. 

MRGxE adopts a gene–covariate interaction as an instrument, subsequently placing restrictions on the interaction analogous to the IV assumptions. A suitable interaction GZ is therefore:

GxE1: Associated to the exposure of interest ($\gamma_{3}\neq 0$).

GxE2: Not associated with confounders of the exposure and outcome (GZ ⊥ U).

GxE3: Not associated with the outcome outside of the exposure of interest ($\beta_{4}=0$).

The first assumption is assessed by directly fitting the first-stage model. For the second assumption, it is important to stress that it pertains to the independence of the *interaction* with respect to confounders, and not G and Z individually. The third assumption requires pleiotropic effects remain constant across the population. Variation in pleiotropic effects can be driven by violations of the second assumption, as outlined in the following section.

A value of Z defining a no-relevance group (observed or hypothetical) can be derived as the covariate value$\;Z=z_{X}$ at which G and X are independent, calculating the partial effect of G upon X and rearranging such that:

(3) $$\frac{dX}{dG}=\gamma_{1}+\gamma_{3}Z_{i}=0.$$

This yields the trivial solution

(4) $$z_{X}=-\left(\frac{\gamma_{1}}{\gamma_{3}}\right).$$

Where $z_{X}$ is observed in the population, regressing Y upon G for the subset of participants with$\;Z=z_{X}$ provides a pleiotropy estimate (that is for$\;\beta_{2})$ as the coefficient of G. Unfortunately, this is difficult to implement in practice, either because the value $z_{X}$ is not observed or the subset of participants is too small to provide sufficient power. Consequently, it is often appropriate to estimate pleiotropy at a theoretical (or extrapolated) no-relevance group, using differences in instrument–exposure associations across Z.

To illustrate how this is possible, a *reduced-form* IV model is constructed—that is, models for X given G, and Y given G by rewriting Model (1) as

(5) $$X_{i}=\gamma_{0}+\left(\gamma_{1}+\gamma_{3}Z_{i}\right)G_{i}+\gamma_{2}Z_{i}+U_{i}+\epsilon_{Xi}$$

and Model (2) as

(6) $$Y_{i}=\beta_{0}+\left[\beta_{1}{(\gamma }_{1}+\gamma_{3}Z_{i})+\beta_{2}+\beta_{4}Z_{i}+U_{i}+\epsilon_{Xi}\right]G_{i}+\beta_{3}Z_{i}+U_{i}+\epsilon_{Yi}.$$

The change in G-X and G-Y associations for a given change in Z can be identified as the coefficient of G in Models (5) and (6), respectively (with$\;\beta_{4}$ set to 0), as

$$G-X\;association:(\gamma_{1}+\gamma_{3}Z_{i}),G-Y\;association:\;[\beta_{1}{(\gamma }_{1}+\gamma_{3}Z_{i})+\beta_{2}].$$

The Wald ratio^24^ estimand for the causal effect of X on Y would then be equal to:

(7) $$\frac{\beta_{1}\left(\gamma_{1}+\gamma_{3}Z_{i}\right)+\beta_{2}}{\gamma_{1}+\gamma_{3}Z_{i}}=\;\beta_{1}+\frac{\beta_{2}}{\gamma_{1}+\gamma_{3}Z_{i}}.$$

This gives the causal effect, $\beta_{1}$, plus a non-zero bias term whenever $\beta_{2}$ is non-zero. In the Cho *et al.*^14^ analysis, an estimate for$\;\beta_{1}$ was obtained by performing TSLS regression using the interaction as the instrument; fitting Models (8) and (9) below:

(8) $$X_{i}=\left(\gamma_{1}+\gamma_{3}Z_{i}\right)G_{i}+\gamma_{2}Z_{i}+\epsilon_{Xi},$$

(9) $$Y_{i}=\beta_{0}+\beta_{1}\hat{X_{i}}+\beta_{2}G_{i}+\beta_{3}Z_{i}+\epsilon_{Yi},$$

where $\hat{X_{i}}$ is the fitted value from Model (8). In this case, the coefficient $\beta_{2}$ represents the degree of pleiotropy for the genetic instrument G.

Whilst this approach does not require an observed no-relevance group, it has two limitations. First, as a consequence of utilizing TSLS, it is restricted to individual-level data. Second, it assumes an underlying linear model, which may not hold in practice. For example, if considering adiposity as an exposure, individuals at extreme values could be at greater risk, implying a curved relationship.

MRGxE overcomes these limitations by reframing the model within a two-sample summary MR context, and executing the following three-step procedure:

1. Estimate G-X and G-Y associations at a range of values of Z.
2. Regress the G-Y associations on the G-X associations within a linear regression.
3. Estimate the causal effect $\beta_{1}$ as the slope of the regression, and the mean pleiotropic effect as the intercept of the regression.

Let$\;Z_{j}$ denote the $j^{th}$ subgroup of Z (j=1,…,J). For each group$\;Z_{j}$, we estimate the instrument–exposure association and standard error (Step 1) using the following regression model:

(10) $$X_{i}=\gamma_{j0}+\gamma_{j1}G_{i}+\epsilon_{jXi}.$$

Note that we include a subscript j to distinguish the regression parameters from the first-stage Model (1). The coefficient $\gamma_{j1}$ is therefore interpreted as the G-X association for group$\;Z_{j}$. Next, we fit the corresponding instrument–outcome regression model (Step 2):

(11) $$Y_{i}=\delta_{0j}+\delta_{j1}G_{i}+\epsilon_{jYi}.$$

We use$\;\delta_{j1}$ to denote the G-Y association coefficient for group$\;Z_{j}$, distinguishing Model (11) from Model (6). Thus, from Models (10) and (11), we obtain sets of G-X associations$\;({\hat{\gamma }}_{J1})$ and G-Y associations$\;({\hat{\delta }}_{J1})$ across$\;Z_{J}$ subgroups. Finally, we regress the set of ${\hat{\delta }}_{J1}$ estimates upon the set of$\;{\hat{\gamma }}_{J1}$ estimates (Step 3):

(12) $${\hat{\delta }}_{J1}={\beta_{GxE}}_{0}+{\beta_{GxE}}_{1}{\hat{\gamma }}_{J1}+\epsilon_{JGxE}.$$

In Model (12), ${\beta_{GxE}}_{0}$ is the pleiotropy estimate $\left(\beta_{2}\right)$, whilst ${\beta_{GxE}}_{1}$ is the effect of X upon Y correcting for pleiotropy ($\beta_{1}$). To illustrate, recall that$\;\beta_{2}$ represents a constant pleiotropic effect across Z. Model (12) is an average of the ratio estimates across $Z_{J}$, with the bias parameter $\beta_{2}$ estimated as the intercept. A diagram illustrating these features is given in the Supplementary Material, available as Supplementary data at *IJE* online, accompanied by a demonstration of how the functional form of the interaction can be inferred from the distribution of the subgroup estimates.

To show how the intercept estimates$\;\beta_{2}$, consider the reduced-form Model (6) evaluated for the no-relevance group $Z=z_{X}$. From Equation (4), $z_{X}=-\left(\frac{\gamma_{1}}{\gamma_{3}}\right)$. Then, by substitution:

(13) $$\beta_{1}\gamma_{1}+{\beta_{1}\gamma }_{3}\left(\frac{{-\gamma }_{1}}{\gamma_{3}}\right)+\beta_{2}=\beta_{1}\gamma_{1}-{\beta_{1}\gamma }_{1}+\beta_{2}=\;\beta_{2}.$$

Where the intercept is zero, the MRGxE causal-effect estimate is identical to an IVW estimate using the subgroup ratio estimates. This mirrors the equivalence of IVW and MR-Egger regression in the multiple instrument setting with balanced pleiotropy. R code for implementing MRGxE is provided in the Supplementary Material, available as Supplementary data at *IJE* online.

Before continuing, it is important to highlight several important factors to consider when implementing MRGxE. Initially, it is important to define an appropriate number of Z subgroups so as to accurately characterize the underlying gene–covariate interaction. Second, it is important to *not* transform effects to be positive, as performed for MR-Egger regression. This mischaracterizes the interaction term, attenuating causal-effect estimates. Finally, where instrument–exposure associations are present for all groups in the same direction, the accuracy in extrapolating the regression line towards a theoretical no-relevance group will be a function of the distance from the minimum $Z_{j}$ instrument–exposure association and variation in the set of $Z_{j}$ instrument–exposure associations. Further guidance and illustrations of these features of MRGxE are presented in the Supplementary Material, available as Supplementary data at *IJE* online.

### The constant-pleiotropy assumption

As a single (constant) parameter,${\;\beta }_{2}$ equates to the ‘correct’ intercept for MRGxE—that is, the intercept that must be estimated in order to identify the correct causal effect ${\;\beta }_{1}.$ Consistent estimates for both${\;\beta }_{1}$ and$\;\beta_{2}$ are produced in cases where the pleiotropic effect remains constant across all values of Z (${\;\beta }_{4}=0$). If${\;\beta }_{4}\neq 0$, then the constant-pleiotropy assumption is violated, leading to the true pleiotropic effect$\;\beta_{2}$ being equated to $\beta_{2}-\frac{\beta_{4}\gamma_{1}}{\gamma_{3}}$ and bias in the causal estimand for${\;\beta }_{1}$ such that:

(14) $${\hat{\beta }}_{1}=\beta_{1}+\frac{\beta_{4}}{\gamma_{3}}.$$

The derivation of this result is provided in the Supplementary Material, available as Supplementary data at *IJE* online. From Equation (14), it is clearly possible to mitigate such bias when the instrument–covariate interaction$\;(\gamma_{3})$ is large relative to the variation in the pleiotropic effect$\;\beta_{4}$, with the bias tending towards zero as$\;\gamma_{3}$ increases. However, as it is not possible to directly estimate $\beta_{4}$, justifying the relative effect sizes of the first- and second-stage interactions requires a priori knowledge.

**Figure 2.**
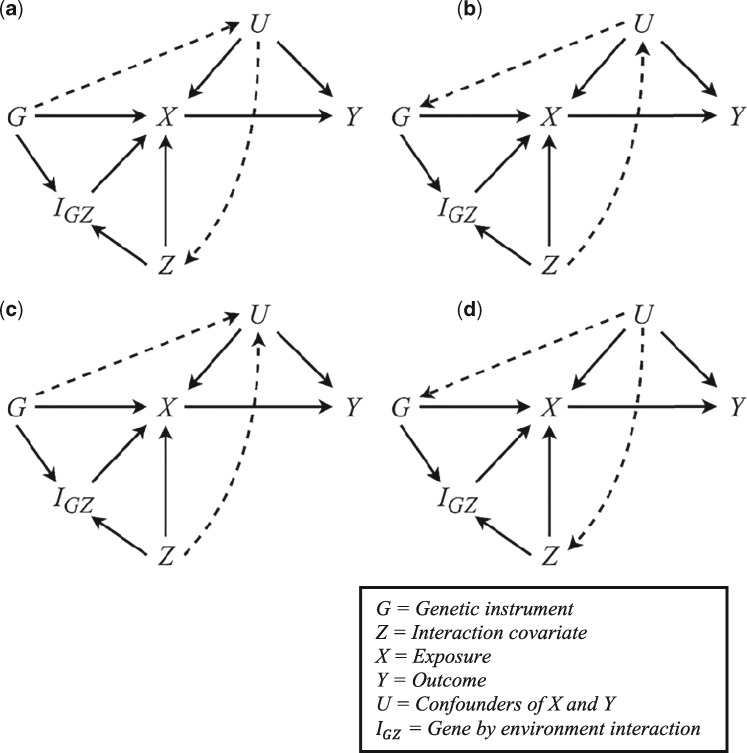
A set of DAGs illustrating interaction-covariate confounding structures indicated by dashed lines. Scenarios (a), (b) and (d) induce bias in MRGxE estimates. However, this is not the case for Scenario (c) or when the confounder is associated with either G or Z individually.

Violations of the constant-pleiotropy assumption can result from specific confounding structures in the underlying true model. Specifically, there must be no downstream pathway from G to Z via the confounders U, no pathway from Z to G through U, and U cannot be a joint determinant of G and Z. Figure 2 shows four possible scenarios in which G and Z are associated with U.

In Figure 2, Scenarios (a), (b) and (d) introduce bias in MRGxE estimates, whilst Scenario (c) and individual associations between either Z and G with U do not. Further details on the underlying mechanisms behind such bias are presented in the Supplementary Material, available as Supplementary data at *IJE* online. As a consequence, the range of interaction covariates suitable for use within MRGxE is not as restrictive as one might naively assume. In an MR context, there are limited cases in which a confounder will be a determinant of a genetic instrument, and this is only problematic where the confounder is simultaneously associated with the interaction covariate. It seems that MRGxE estimates will be most susceptible to bias where the instrument is a determinant of one or more confounders, which in turn are determinants of the interaction covariate. We recommend care be taken in examining such pathways and suggest MRGxE be implemented as one component of a series of sensitivity analyses, as with other such approaches.^4^

### MRGxE as a sensitivity analysis

In cases where the constant-pleiotropy assumption is assumed to be violated, MRGxE can still be applied in sensitivity analyses to select a subset of valid instruments. To demonstrate, consider that an invalid instrument can be detected, in principle whenever $\beta_{2}-\frac{\beta_{4}\gamma_{1}}{\gamma_{3}}\;\neq 0$, due to either$\;\beta_{2}\neq 0$,$\;\beta_{4}\neq 0$ or both. Consequently, MRGxE can be used to assess the validity of individual instruments, informing instrument selection and evaluating the appropriateness of their incorporation in allelic scores. There are, however, two important considerations when applying this approach. First, it is not possible to distinguish the average pleiotropic effect across interaction-covariate subgroups ($\beta_{2}$) from the change in pleiotropic effect between instrument–covariate subgroups $\left(\beta_{4}\right)$. It is therefore a test of invalidity due to either factor and cannot be used to correct MRGxE estimates directly. Second, MRGxE will incorrectly fail to detect invalid instruments (a Type II error) in the special case where $\beta_{2}$ is close to $\left(-\frac{\beta_{4}\gamma_{1}}{\gamma_{3}}\right)$.

### Causal effect of BMI upon SBP

Previous observational,^25^ randomized control trials^26^ and MR^27–29^ studies have reported evidence of a positive association between BMI and SBP. However, the magnitude of this association differs markedly between such studies, with observational studies often recording greater effect sizes than those using MR.

As an applied example, we perform two-sample summary MR and MRGxE analyses examining the effect of BMI upon SBP using variants identified from the GIANT consortium^22^ and two non-overlapping random samples of UK Biobank. The decision to use two subsamples of the UK Biobank, as opposed to summary estimates from the GIANT consortium, is motivated by potential differences in the standardization of BMI between each sample. As MRGxE utilizes BMI values from the UK Biobank, selecting two subsamples for which BMI has been identically standardized allows a more effective comparison of the approaches.

The purpose of performing both two-sample summary and MRGxE analyses is to highlight the extent to which pleiotropic effect estimates obtained using MRGxE with a single instrument agree with conventional MR approaches. Initially, the UK Biobank sample contained a total of 502 614 individuals. From this sample, we excluded participants who failed to meet quality control, specifically in cases where genetic and reported sex conflicted, where sex chromosome karyotypes were putatively different from XX and XY, and individuals who were outliers with respect to heterozygosity and missing rates. Further, we removed participants of non-European ancestry and related individuals by preferentially removing individuals related to the greatest number of individuals until no related pairs remained. This resulted in a total of 358 928 participants being included in the analyses.

In conducting a two-sample summary analysis, effect estimates and standard errors for 96 genetic variants identified by the GIANT consortium as being robustly associated with BMI $\left(p=5\times 10^{-8}\right)$ were obtained from a 50% random sample of the UK Biobank.^22^ Corresponding estimates for each genetic variant with respect to SBP were obtained using the remaining UK Biobank sample. In contrast, MRGxE was implemented by constructing a weighted allelic score informed using estimates from the GIANT consortium. The MRGxE analysis can be viewed as analogous to two-sample summary MR, using instrument–exposure estimates for BMI as external weights and individual data from a separate sample to inform instrument–outcome association estimates. In each analysis, BMI, SBP and the weighted allelic score were standardized using a z-score transformation.

### Two-sample summary analyses

We implement several two-sample summary MR methods utilizing the mrrobust software package^30^ in Stata SE 14.0.^31^ IVW provides estimates with greater precision than alternative summary approaches; however, as such estimates can exhibit bias in the presence of pleiotropy, MR-Egger regression, weighted median and weighted modal approaches are implemented as sensitivity analyses.

A range of methods are adopted in sensitivity analyses, as each method relies upon differing assumptions with respect to the underlying distribution of pleiotropic effects. MR-Egger regression requires the effect of genetic variants on the exposure to be independent of their pleiotropic effects on the outcome (InSIDE).^5^ The weighted median requires more than 50% of the variants to be valid instruments accounting for weighting,^7^ whilst the modal estimator assumes that the most frequent value of the pleiotropic bias across the set of genetic variants is zero (ZEMPA).^32^

### MRGxE analyses using Townsend Deprivation Index

In implementing MRGxE, Townsend Deprivation Index (TDI) was selected as a continuous covariate for which instrument strength varies, based on findings from previous studies.^16^^,^^33^ TDI is a common derived measure of socio-economic deprivation, using many variables such as car ownership, occupation type and educational attainment.^34^ It is measured at an area level (electoral wards), with participants assigned a score based upon the area in which they lived.^34^ Missing values were removed prior to performing the analysis, with observational and TSLS estimates presented in the Supplementary Material, available as Supplementary data at *IJE* online.

### Simulation overview

To illustrate the effectiveness of MRGxE, and further consider the importance of the constant-pleiotropy assumption with respect to causal-effect estimation, we performed a simulation study within a two-sample MR framework. Considering a realistic case, two sets of simulations were performed, the first using a null causal effect$\;(\beta_{1}=0$) and indexed as A, and the second a positive causal effect$\;(\beta_{1}=0.05$) indexed as B. Individual-level data are generated, from which the necessary summary-data estimates are extracted. In each case, a total of 5 population subgroups are used from a sample size of 50 000, with further details provided in the Supplementary Material, available as Supplementary data at *IJE* online.

Four distinct cases were considered:

Case 1: No pleiotropy and the constant-pleiotropy assumption satisfied

Case 2: Directional pleiotropy and the constant-pleiotropy assumption satisfied

Case 3: No pleiotropy and the constant-pleiotropy assumption violated

Case 4: Directional pleiotropy and the constant-pleiotropy assumption violated

The results for each case represent the mean values for 10 000 simulated datasets.

## Results

### Analysis I: two-sample summary analysis

Estimates obtained from implementing each two-sample summary MR approach are presented in Table 1. All of the methods performed with the exception of SIMEX-corrected MR-Egger show evidence of a positive association between BMI and SBP. There also appears limited evidence of a pleiotropic effect, with the IVW estimate lying within the confidence intervals of both the weighted median and weighted modal estimates.

**Table 1.** Two-sample summary MR estimates for the effect of body mass index (BMI) upon systolic blood pressure (SBP). A smoothing parameter (ϕ=1) was selected in implementing the modal estimator and a value $I_{GX}^{2}=0.89$ using MR-Egger is indicative of regression dilution of approximately 11% towards the null

Method	Estimate	SE	95% CI	*p*-value
IVW	0.101	0.031	(0.04, 0.16)	0.001
MR-Egger (intercept) MR-Egger (effect)^a^	0.002 0.027	0.001 0.062	(–0.001, 0.005) (–0.09, 0.15)	0.173 0.658
SIMEX-corrected MR-Egger (intercept) SIMEX-corrected MR-Egger (effect)	0.003 –0.020	0.003 0.154	(–0.003, 0.01) (–0.32, 0.28)	0.898 0.325
Weighted median	0.147	0.032	(0.08, 0.21)	<0.001
Modal estimator^b^	0.102	0.031	(0.04, 0.16)	0.001

a $I_{GX}^{2}=0.90$

b Smoothing parameter ϕ=1.

### Analysis II: MRGxE using TDI

To perform MRGxE, we divided the sample using quantiles of TDI into 5, 10, 20 and 50 population subgroups, after which IVW and MRGxE estimates were produced. The results of each analysis are presented in Table 2, with IVW referring to an inverse-variance-weighted estimate using interaction-covariate subgroups.

**Table 2.** Inverse-variance-weighted (IVW) and MRGxE estimates for different numbers of Townsend Deprivation Index (TDI) quantile groupings. The IVW estimate represents an inverse weighted estimate using each of the TDI subgroups, providing an estimate equivalent to two-stage least-squares estimates using the weighted allelic score

Number of groups	Method	Estimate	SE	95% CI	*p*-value
	MRGxE (intercept)	–0.007	0.007	(–0.03, 0.02)	0.383
5	MRGxE (effect)	0.161	0.054	(–0.01, 0.33)	0.059
	IVW	0.106	0.008	(0.08, 0.13)	0.0002
	MRGxE (intercept)	–0.009	0.009	(–0.03, 0.01)	0.347
10	MRGxE (effect)	0.169	0.064	(0.02, 0.32)	0.030
	IVW	0.106	0.010	(0.08, 0.13)	<0.0001
	MRGxE (intercept)	–0.005	0.012	(–0.03, 0.02)	0.669
20	MRGxE (effect)	0.144	0.088	(–0.04, 0.33)	0.121
	IVW	0.106	0.013	(0.08, 0.13)	<0.0001
	MRGxE (intercept)	–0.007	0.011	(–0.03, 0.01)	0.517
50	MRGxE (effect)	0.157	0.078	(0.000, 0.31)	0.049
	IVW	0.107	0.013	(0.08, 0.13)	<0.0001

The estimates in Table 2 largely agree with the two-sample summary MR estimates in several aspects, with the direction of effect remaining consistent across each of the methods applied. This again implies a positive effect of BMI upon SBP. Considering the MR-Egger and MRGxE intercept estimates, there also appears to be little evidence of substantial pleiotropic bias. Constraining the MRGxE model to the intercept yields an estimate equivalent to the two-sample summary IVW estimate presented in Table 1.

Notably, whilst the MR-Egger estimates are consistent with the MRGxE estimates, the effects are markedly different, with MRGxE returning a positive point estimate greater in magnitude than the IVW estimate. The difference in these estimates can be attributed to the differing intercept estimates that, while close to zero in both cases, are different in terms of direction. As the MR-Egger and MRGxE intercept estimates lie within their overlapping confidence intervals, we would highlight this as a case where the discrepancy may be due to a lack of precision. In this case, it could be argued to be appropriate to conclude that there is a lack of robustly identified directional pleiotropic effect and adopt the IVW estimate.

**Figure 3.**
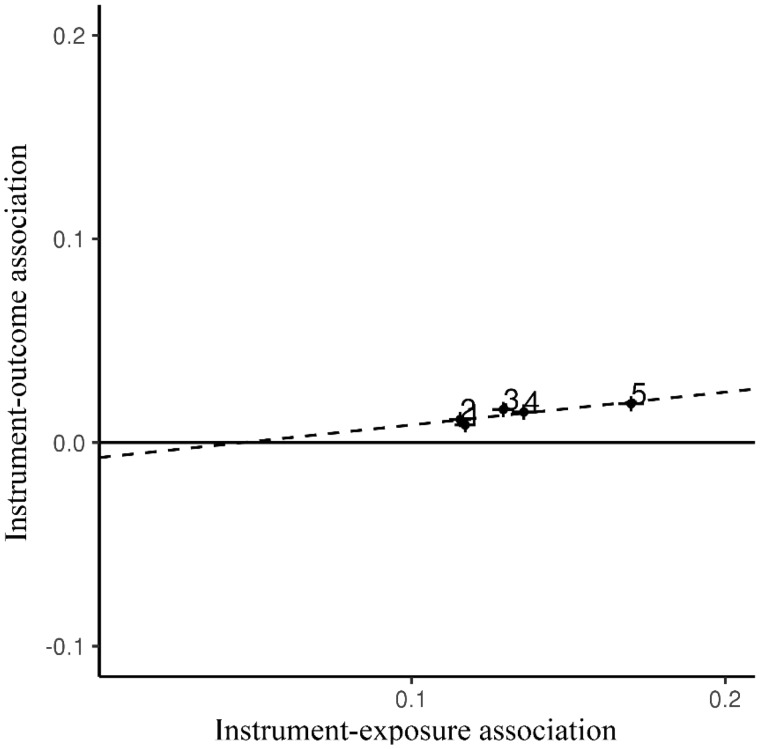
A scatterplot showing the MRGxE estimate indicated as a dashed line. Each point represents ascending quintiles of the Townsend Deprivation Index, in this case showing the strength of the instrument–exposure association to increase with increasing socio-economic deprivation.

Figure 3 displays both the IVW and MRGxE estimates for the five-group case, whilst corresponding plots for other groups are presented in the Supplementary Material, available as Supplementary data at *IJE* online.

Considering Figure 3, the ordering of the TDI groups supports the assumption that the instrument–exposure association varies across levels of TDI. In particular, the least deprived groups (Group 1 and Group 2) have the weakest association, suggesting that genetically predicted BMI is a weaker predictor of BMI for participants experiencing lower levels of deprivation. A further observation is that the positioning of each estimate provides some evidence of a linear interaction, with instrument strength increasing monotonically as subgroup TDI increases. However, the close proximity of Groups 1 and 2, as well as Groups 3 and 4, could be indicative of non-linearity, as they could represent inflection points in the underlying distribution of the interaction (see the Supplementary Material, available as Supplementary data at *IJE* online, for inference guidelines).

One important consideration in performing MR analyses is that causal-effect estimates are often uncertain, due to either a lack of precision or doubts regarding the assumptions of the approach. One response put forward by VanderWeele *et al.*^35^ has been to shift the emphasis from identifying the magnitude of causal effects to identifying the presence of causal effects. Under such a paradigm, estimation using instrument–covariate interactions, such as through MRGxE, can be insightful in identifying broad effects or associations. Adopting this rationale, MRGxE can be used as a broad test of instrument validity in cases where the underlying assumptions of the approach are likely violated, focusing on effect direction as opposed to effect magnitude.

### Simulations

Results of the simulation analyses are presented in Table 3. The mean F statistic remains the same for each case, with substantial variation in the F statistic between interaction-covariate groups. This is essential, as the variation in instrument strength is representative of variation in instrument relevance across population subgroups. Estimates using IVW and MRGxE, as well as significance values, were taken directly from each regression output without using regression weights, as the variant–outcome associations have the same standard errors.

**Table 3.** Performance of IVW and MRGxE methods in simulation setting. In Case 4, $\beta_{4}=\beta_{2}$ and consequentially the intercept of the MRGxE model is approximately 0

Case	IVW Mean estimate (mean SE)	IVW Type I error rate	MRGxE Mean estimate (mean SE)	MRGxE Power of pleiotropy test	MRGxE Effect Type I error rate
Case 1A:	0.000 (0.021)	0.050	0.001 (0.030)	0.049	0.052
B	0.050 (0.021)	0.423	0.051 (0.030)	0.049	0.207
Case 2A:	0.088 (0.046)	0.135	0.001 (0.030)	0.708	0.052
B	0.138 (0.046)	0.630	0.051 (0.030)	0.708	0.207
Case 3A:	0.079 (0.046)	0.091	0.167 (0.027)	0.770	0.945
B	0.129 (0.046)	0.534	0.217 (0.027)	0.770	0.993
Case 4A:	0.167 (0.019)	1.000	0.167 (0.027)	0.047	0.945
B	0.217 (0.019)	1.000	0.217 (0.027)	0.047	0.993

In the valid instrument case, both IVW and MRGxE provide unbiased effect estimates, though the IVW estimate is more accurate. This is similar to comparisons between IVW and MR-Egger regression, supporting use of IVW in cases where pleiotropy is absent. Type I error rates remained at approximately 5% for both IVW and MRGxE. In the second case, IVW exhibits bias, whilst MRGxE continues to produce unbiased estimates.

In the third case, the instrument is not valid, but pleiotropic effects change across population subgroups. Here, both IVW and MRGxE produce biased causal-effect estimates, with the MRGxE effect estimates showing a greater degree of bias than the IVW estimates. This increases the Type I error rate relative to IVW. In this situation, the MRGxE test for pleiotropy is particularly powerful, though this seeming increase in power can be attributed to violation of the constant-pleiotropy assumption $\left(\beta_{4}\neq 0\right)$ leading to over-estimation of the magnitude of the pleiotropic effects. In the final case, both IVW and MRGxE produce estimates with similar bias and precision. Here, the MRGxE test for pleiotropy is suggestive of a null pleiotropic effect, remaining at 5%. This represents a situation in which $\beta_{2}=\beta_{4}$, invalidating the use of MRGxE as a sensitivity analysis.

## Discussion

In this paper, we present MRGxE as a simple and intuitive method to identify and correct for pleiotropic bias in MR studies using instrument–covariate interactions. MRGxE enables the pleiotropic effect of individual instruments (or single allele scores) to be assessed and, when such pleiotropy exists and satisfies the constant-pleiotropy assumption, MRGxE provides improved causal estimation compared with IVW. In the absence of such pleiotropy, the IVW approach is more accurate and should be preferred.

In cases where the constant-pleiotropy assumption is violated, a sensible approach would be to prune invalid variants using pleiotropy estimates from MRGxE and then implement IVW using valid variants. In this sense, MRGxE can be viewed very much as a tool for sensitivity analysis.

### Two-sample summary MRGxE

Whilst this paper has focused on the application of MRGxE to individual-level data (albeit by extracting and then meta-analysing summary statistics obtained from it), it clearly applies where interaction–subgroup-specific summary data on instrument–exposure and instrument–outcome associations are available. An alternative approach would be to meta-analyse summary statistics obtained from many separate studies under the assumption that study-specific estimates relate to a study-specific characteristic. For example, the work of Robinson *et al.*^36^ highlights the interaction between age and adult BMI heritability as one potential candidate, given that age is likely to vary naturally across contributing studies.

### Limitations of MRGxE

A number of factors must be considered before implementing MRGxE. First, the constant-pleiotropy assumption is essential for causal estimate correction. If there is reason to believe that pleiotropic effects differ between population subgroups, then the approach will give misleading effect estimates. One useful aspect to this problem, however, is that, provided the first-stage interaction is sufficiently strong, bias from changes in pleiotropic effect may be sufficiently small as to be negligible in analyses. This may well be the case in situations such as the Cho *et al.*^14^ study, where the difference in instrument effect between gender groups is very strong in comparison to potential variation in pleiotropic effect. In our analyses, the use of an allele score as a single (strong) instrument meant that it was naturally much more robust to bias than any individual component SNP. One strategy to overcome this limitation would be to carry out several analyses using differing interaction covariates. Provided that the instrument–covariate interaction of sufficient strength, it would be expected that resulting estimates would be in agreement. In cases where substantial disagreement is observed, such disagreement could be indicative of violation of the constant-pleiotropy assumption or characteristics of the underlying confounding structure. The work of Emdin *et al.*^15^ and Krishna *et al.*^37^ follows this reasoning. Further work will consider the implications of interaction-covariate selection and the role of confounding within the context of MRGxE.

A second limitation is that, owing to the limited availability of summary-data estimates for particular covariate groups, it may be difficult to implement in a summary-data setting. At present, researchers may be limited to common groupings such as gender.

Finally, it is important to consider results from MR gene–environment interaction approaches within the context of existing evidence using alternate estimation approaches, within the triangulation framework^38^^,^^39^ in which differences in estimates across a range of approaches can be indicative of sources of bias potentially unique to each research design. Identifying disagreement in estimated effects across studies of differing design can therefore prove valuable in identifying avenues for further research, whilst substantial agreement strengthens confidence in the resulting findings and subsequent inference.

## Funding

This work was supported by the Medical Research Council (MRC) and the University of Bristol, who fund the MRC Integrative Epidemiology Unit (MC_UU_00011/1, MC_UU_00011/2).

Wes Spiller is supported by a Wellcome Trust studentship (108902/B/15/Z).

## Supplementary Material

dyy204_Supplementary_Data

## References

[1] Davey Smith G, Ebrahim S. ‘ Mendelian randomization’: can genetic epidemiology contribute to understanding environmental determinants of disease? Int J Epidemiol 2003;32:1–22. 12689998 10.1093/ije/dyg070

[2] Davey Smith G, Hemani G. Mendelian randomization: genetic anchors for causal inference in epidemiological studies. Hum Mol Genet 2014;23:R89–98. 25064373 10.1093/hmg/ddu328 PMC4170722

[3] Burgess S, Small DS, Thompson SG. A review of instrumental variable estimators for Mendelian randomization. Stat Methods Med Res 2017;26:2333–55. 26282889 10.1177/0962280215597579 PMC5642006

[4] Hemani G, Bowden J, Davey Smith G. Evaluating the potential role of pleiotropy in Mendelian randomization studies. Hum Mol Genet 2018;27:R195–208. 29771313 10.1093/hmg/ddy163 PMC6061876

[5] Bowden J, Davey Smith G, Burgess S. Mendelian randomization with invalid instruments: effect estimation and bias detection through Egger regression. Int J Epidemiol 2015;44:512–25. 26050253 10.1093/ije/dyv080 PMC4469799

[6] Burgess S, Thompson SG. Multivariable Mendelian randomization: the use of pleiotropic genetic variants to estimate causal effects. Am J Epidemiol 2015;181:251–60. 25632051 10.1093/aje/kwu283 PMC4325677

[7] Bowden J, Davey Smith G, Haycock PC, Burgess S. Consistent estimation in Mendelian randomization with some invalid instruments using a weighted median estimator. Genet Epidemiol 2016;40:304–14. 27061298 10.1002/gepi.21965 PMC4849733

[8] Hartwig FP, Davey Smith G, Bowden J. Robust inference in summary data Mendelian randomization via the zero modal pleiotropy assumption. Int J Epidemiol 2017;46:1985–98. 29040600 10.1093/ije/dyx102 PMC5837715

[9] Slichter D. Testing Instrument Validity and Identification with Invalid Instruments. Rochester: University of Rochester, 2014.

[10] Imbens GW, Angrist JD. Identification and estimation of local average treatment effects. Econometrica 1994;62:467–75.

[11] Card D. Using Geographic Variation in College Proximity to Estimate the Return to Schooling. Cambridge, Massachusetts: National Bureau of Economic Research, Inc, 1993.

[12] Conley TG, Hansen CB, Rossi PE. Plausibly exogenous. Rev Econ Stat 2012;94:260–72.

[13] Small DS. Mediation analysis without sequential ignorability: using baseline covariates interacted with random assignment as instrumental variables. J Stat Res 2012;46:91–103. PMC4244702 25435642

[14] Cho Y, Shin SY, Won S, Relton CL, Davey Smith G, Shin MJ. Alcohol intake and cardiovascular risk factors: A Mendelian randomisation study. Sci Rep 2015;5:18422. 26687910 10.1038/srep18422 PMC4685310

[15] Emdin CA, Khera AV, Natarajan P et al Genetic association of waist-to-hip ratio with cardiometabolic traits, type 2 diabetes, and coronary heart disease. JAMA 2017;317:626–34. 28196256 10.1001/jama.2016.21042 PMC5571980

[16] Tyrrell J, Wood AR, Ames RM et al Gene-obesogenic environment interactions in the UK Biobank study. Int J Epidemiol 2017;46:559–75. 28073954 10.1093/ije/dyw337 PMC5837271

[17] Davey Smith G. Use of genetic markers and gene-diet interactions for interrogating population-level causal influences of diet on health. Genes Nutr 2011;6:27–43. 21437028 10.1007/s12263-010-0181-y PMC3040803

[18] Lewis SJ, Davey Smith G. Alcohol, ALDH2, and esophageal cancer: a meta-analysis which illustrates the potentials and limitations of a Mendelian randomization approach. Cancer Epidemiol Biomarkers Prev 2005;14:1967–71. 16103445 10.1158/1055-9965.EPI-05-0196

[19] Chen L, Davey Smith G, Harbord RM, Lewis SJ. Alcohol intake and blood pressure: a systematic review implementing a Mendelian randomization approach. Plos Med 2008;5:e52–71. 18318597 10.1371/journal.pmed.0050052 PMC2265305

[20] Davey Smith G. Mendelian randomization for strengthening causal inference in observational studies: application to gene x environment interactions. Perspect Psychol Sci 2010;5:527–45. 26162196 10.1177/1745691610383505

[21] van Kippersluis H, Rietveld CA. Pleiotropy-robust Mendelian randomization. Int J Epidemiol 2017;47:1279–88. 10.1093/ije/dyx002 PMC6124631 28338774

[22] Locke AE, Kahali B, Berndt SI et al Genetic studies of body mass index yield new insights for obesity biology. Nature 2015;518:197–206. 25673413 10.1038/nature14177 PMC4382211

[23] Taylor AE, Lu F, Carslake D et al Exploring causal associations of alcohol with cardiovascular and metabolic risk factors in a Chinese population using Mendelian randomization analysis. Sci Rep 2015;5:14005. 26364564 10.1038/srep14005 PMC4568464

[24] Wald A. The fitting of straight lines if both variables are subject to error. Ann Math Stat 1940;11:284–300.

[25] Droyvold WB, Midthjell K, Nilsen TI, Holmen J. Change in body mass index and its impact on blood pressure: a prospective population study. Int J Obes (Lond) 2005;29:650–55. 15809666 10.1038/sj.ijo.0802944

[26] Neter JE, Stam BE, Kok FJ, Grobbee DE, Geleijnse JM. Influence of weight reduction on blood pressure: a meta-analysis of randomized controlled trials. Hypertension 2003;42:878–84. 12975389 10.1161/01.HYP.0000094221.86888.AE

[27] Li LJ, Liao J, Cheung CY et al Assessing the causality between blood pressure and retinal vascular caliber through Mendelian randomisation. Sci Rep 2016;6:22031. 26911737 10.1038/srep22031 PMC4766565

[28] Timpson NJ, Harbord R, Davey Smith G, Zacho J, Tybjaerg-Hansen A, Nordestgaard BG. Does greater adiposity increase blood pressure and hypertension risk? Mendelian randomization using the FTO/MC4R genotype. Hypertension 2009;54:84–90. 19470880 10.1161/HYPERTENSIONAHA.109.130005

[29] Holmes MV, Lange LA, Palmer T et al Causal effects of body mass index on cardiometabolic traits and events: a Mendelian randomization analysis. Am J Hum Genet 2014;94:198–208. 24462370 10.1016/j.ajhg.2013.12.014 PMC3928659

[30] Spiller W, Davies NM, Palmer TM. Software application profile: mrrobust—a tool for performing two-sample summary Mendelian randomization analyses. Int J Epidemiol 2019 48:684–90.

[31] StataCorp. Stata Statistical Software: Release 14. College Station, TX: StataCorp LP, 2015.

[32] Hartwig FP, Davey Smith G, Bowden J. Robust inference in summary data Mendelian randomization via the zero modal pleiotropy assumption. Int J Epidemiol 2017;46:1985–98. 29040600 10.1093/ije/dyx102 PMC5837715

[33] Rask-Andersen M, Karlsson T, Ek WE, Johansson Å. Gene-environment interaction study for BMI reveals interactions between genetic factors and physical activity, alcohol consumption and socioeconomic status. PLOS Genet 2017;13:e1006977. 28873402 10.1371/journal.pgen.1006977 PMC5600404

[34] Mackenbach JP. Health and deprivation—inequality and the North In: Townsend P, Phillimore P, Beattie A (eds). Health Policy, Vol. 10. London: Routledge, 1988, p. 207.

[35] VanderWeele TJ, Tchetgen Tchetgen EJ, Cornelis M, Kraft P. Methodological challenges in Mendelian randomization. Epidemiology 2014;25:427–35. 24681576 10.1097/EDE.0000000000000081 PMC3981897

[36] Robinson MR, English G, Moser G et al Genotype-covariate interaction effects and the heritability of adult body mass index. Nat Genet 2017;49:1174–81. 28692066 10.1038/ng.3912

[37] Krishna A, Razak F, Lebel A, Davey Smith G, Subramanian SV. Trends in group inequalities and interindividual inequalities in BMI in the United States, 1993–2012. Am J Clin Nutr 2015;101:598–605. 25733645 10.3945/ajcn.114.100073

[38] Munafo MR, Davey Smith G. Robust research needs many lines of evidence. Nature 2018;553:399–401. 29368721 10.1038/d41586-018-01023-3

[39] Lawlor DA, Tilling K, Davey Smith G. Triangulation in aetiological epidemiology. Int J Epidemiol 2016;45:1866–86. 28108528 10.1093/ije/dyw314 PMC5841843

