# Supplementary material for: Detecting and correcting for bias in Mendelian randomization analyses using Gene-by-Environment interactions
Source: Int J Epidemiol. 2018 Nov 20;48(3):702–12. doi: 10.1093/ije/dyy204 (PMC6659360; doi:10.1093/ije/dyy204)
Supplement: dyy204_Supplementary_Data [file dyy204_supplementary_data.docx]

**Supplementary Material (updated 27/07/18)**

Contents

[Simulation demonstrating impact of pleiotropic bias on OLS and IV estimation 2](#_Toc520467937)

[Diagram illustrating features of MRGxE 3](#_Toc520467938)

[Simulations highlighting features of subgroup selection 4](#_Toc520467939)

[R code for implementing MRGXE using 5 interaction-covariate groups and a continuous interaction covariate. 8](#_Toc520467940)

[Simulation demonstrating attenuation bias from transforming instrument-exposure associations to be positive 9](#_Toc520467941)

[Simulation demonstrating relationship between pleiotropy estimate precision and distance of extrapolation 12](#_Toc520467942)

[Derivation of the bias induced through violation of the constant pleiotropy assumption ($\boldsymbol{\beta}\boldsymbol{4}\neq\boldsymbol{0}$). 14](#_Toc520467943)

[Derivation of the violations of the constant pleiotropy assumption through differing confounding structures. 17](#_Toc520467944)

[Observational and TSLS estimates using the UK Biobank sample 25](#_Toc520467945)

[Scatter plots for differing group selection in MRGXE applied analysis 26](#_Toc520467946)

[Simulation specifications 28](#_Toc520467947)

# Simulation demonstrating impact of pleiotropic bias on OLS and IV estimation

We simulate a two-stage IV model using a binary instrument $G$, a continuous exposure $X$, a continuous outcome $Y$, and a set of confounding variables$U$, such that the errors from the first and second stage models are correlated (e.g. with correlation coefficient $\rho=0.5$). We used a sample size of 100,000 observations (instrument $F=5855$), with first and second stage models defined as:

$X_{i}=\gamma_{0}+\gamma_{1}G_{i}+U_{i}+\epsilon_{Xi}$ (A1)

$Y_{i}=\beta_{0}+\beta_{1}X_{i}+\beta_{2}G_{i}+U_{i}+\epsilon_{Yi}$ (A2)

In each scenario we vary the degree of horizontal pleiotropy via$\beta_{2}$. In this example, the true effect of$X$ upon$Y$ is defined as 1 with results from each method presented in Table A1.

***Table A1***: Ordinary least squares (OLS) and two-stage least squares (TSLS) estimates under differing degrees of pleiotropy ($\beta_{2}$).

| **Induced Pleiotropy** | **Model** | **Exposure Estimate**$\boldsymbol{\beta}_{\boldsymbol{1}}\boldsymbol{=1}$ | **95%** $\boldsymbol{C}\boldsymbol{I}^{\boldsymbol{1}}$ |
| --- | --- | --- | --- |
| $\boldsymbol{\beta}_{\boldsymbol{2}}\boldsymbol{=0}$ | OLS  TSLS | 1.15  1.02 | (1.14, 1.16)  (0.98, 1.06) |
| $\boldsymbol{\beta}_{\boldsymbol{2}}\boldsymbol{=1}$ | OLS  TSLS | 1.22  1.97 | (1.21, 1.23)  (1.90, 2.04) |

^1^Confidence Interval

From Table A1 we can see that TSLS provides a more accurate estimate in cases where there is no observed directional pleiotropic effect$(\beta_{2}=0)$, with the OLS estimate showing a significant positive bias in comparison to the TSLS estimate. However, when$\beta_{2}\neq0$, as in the above example, the bias in the TSLS estimate is much greater than the OLS estimate. Any remaining bias when $\beta_{2}=0$ can be attributed to weak instrument bias or sampling error.

# Diagram illustrating features of MRGxE


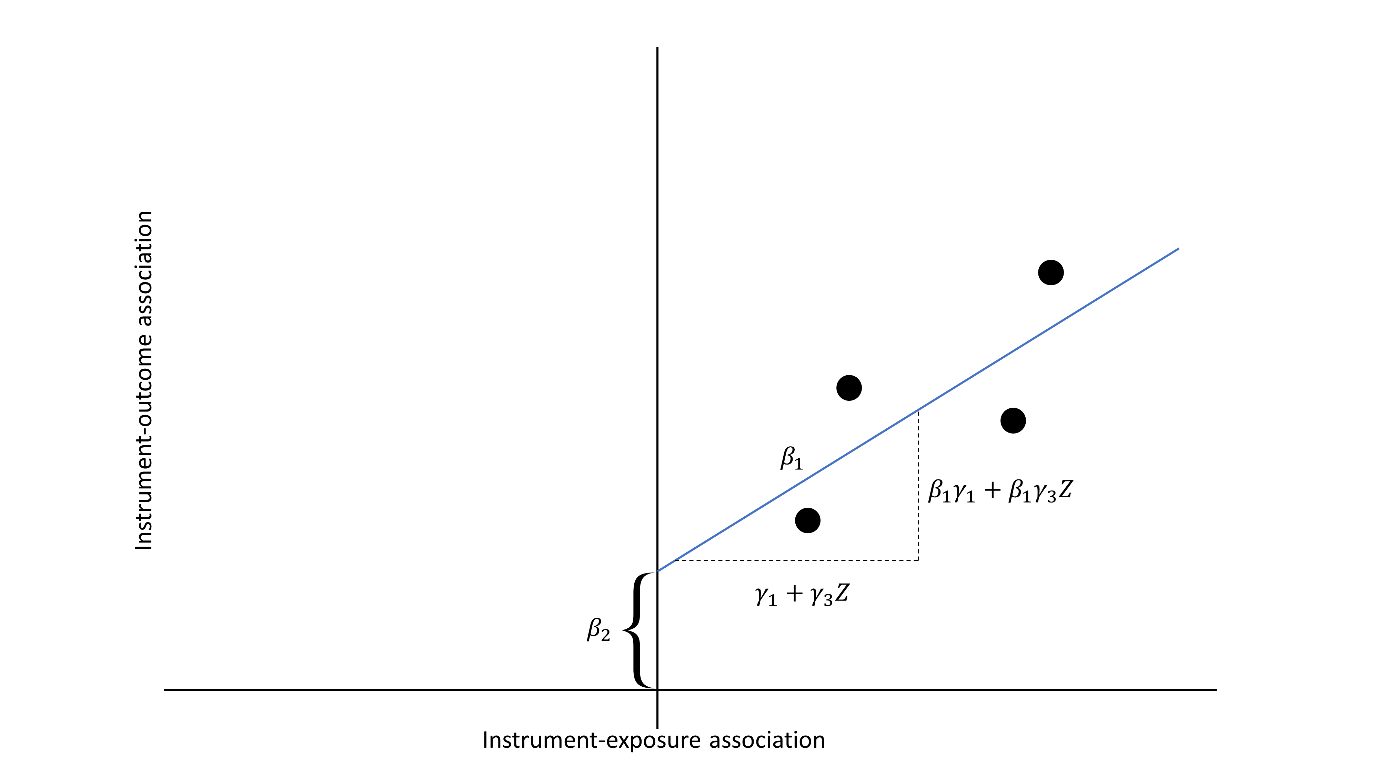


***Figure A1:*** Hypothetical plot showing components of MRGxE. For a set of$Z$ groups represented as solid points, the x-axis represents the association between the genetic instrument and the exposure, whilst the y-axis shows the association between the genetic instrument and the outcome. The point at which $x=0$ is an estimate of the theoretical no relevance group, with a remaining (pleiotropic) association between the instrument and the outcome given as the intercept ($\beta_{2}$).

# Simulations highlighting features of subgroup selection

This simulation has two central aims. Firstly, it seeks to highlight the impact of group number upon association estimates and estimates of instrument validity. Secondly, it illustrates how the functional form of the interaction can be inferred from the distribution of subgroups when plotting MRGXE estimates.

We return to our simulated example above, including a further covariate $Z$ for use as an interaction for MRGXE. In this case, we define three two stage models, using a linear, quadratic, and cubic interaction. The set of two stage models are defined as

Linear

$X_{i}=\gamma_{0}+\gamma_{1}G_{i}+\gamma_{2}Z_{i}+\gamma_{3}{GZ}_{i}+U_{i}+\epsilon_{Xi}$ (A3-1)

$Y_{i}=\beta_{0}+\beta_{1}X_{i}+\beta_{2}G_{i}+\beta_{3}Z_{i}+\beta_{4}GZ_{i}+U_{i}+\epsilon_{Yi}$ (A3-2)

Quadratic

$X_{i}=\gamma_{0}+\gamma_{1}G_{i}+\gamma_{2}Z_{i}+\gamma_{3}{GZ}_{i}^{2}+U_{i}+\epsilon_{Xi}$ (A4-1)

$Y_{i}=\beta_{0}+\beta_{1}X_{i}+\beta_{2}G_{i}+\beta_{3}Z_{i}+\beta_{4}GZ_{i}^{2}+U_{i}+\epsilon_{Yi}$ (A4-2)

Cubic

$X_{i}=\gamma_{0}+\gamma_{1}G_{i}+\gamma_{2}Z_{i}+\gamma_{3}{GZ}_{i}^{3}+U_{i}+\epsilon_{Xi}$ (A5-1)

$Y_{i}=\beta_{0}+\beta_{1}X_{i}+\beta_{2}G_{i}+\beta_{3}Z_{i}+\beta_{4}GZ_{i}^{3}+U_{i}+\epsilon_{Yi}$ (A5-2)

For each model, the causal association between $X$ and $Y$ ($\beta_{1}$), and the direct association between $G$ and $Y$ were set to 0.05. With the exception of $\beta_{4}$ the remaining coefficients were fixed at 1, and a set of confounding variables$U$ was again generated such that the errors from the first and second stage models were correlated (e.g. with correlation coefficient $\rho=0.5$).

Defining too few groups reduces power to detect either an association or instrument invalidity, due to the loss of information when combining two or more distinct groups. Where this is the case an estimate $\hat{\gamma_{j1}}$ represents an average across the two groups, diminishing differences in instrument-exposure association across the set of estimates. Additionally, the lack of observations for instrument-exposure and instrument-outcome associations has an adverse effect on statistical power. The primary concern in selecting too many groups is that as the number of groups increases, the number of observations within each group decreases, reducing the precision of instrument-exposure and instrument-outcome association estimates. We calculated MRGXE estimates using 3 groups, 10 groups, and 100 groups respectively for each two-stage data generating model, with results given in Table A2.

***Table A2***: MRGXE estimates corresponding to models (A3), (A4), and (A5); including power to detect an association or instrument validity.

| **Interaction functional form** | **Number of groups** | **Mean MRGXE estimate** | **Mean MRGXE intercept** | **Power to detect association** | **Power to detect instrument invalidity** |
| --- | --- | --- | --- | --- | --- |
| **linear** | 3 | 0.0507 | 0.0500 | 0.159 | 0.079 |
|  | 10 | 0.0521 | 0.0489 | 0.603 | 0.344 |
|  | 100 | 0.0704 | 0.0305 | 0.931 | 0.208 |
|  |  |  |  |  |  |
| **quadratic** | 3 | 0.0506 | 0.0496 | 0.349 | 0.089 |
|  | 10 | 0.0509 | 0.0492 | 0.766 | 0.193 |
|  | 100 | 0.0602 | 0.0305 | 0.983 | 0.131 |
|  |  |  |  |  |  |
| **cubic** | 3 | 0.0503 | 0.0506 | 0.901 | 0.121 |
|  | 10 | 0.0504 | 0.0506 | 0.999 | 0.544 |
|  | 100 | 0.0518 | 0.0492 | 0.999 | 0.618 |

From Table A2 we demonstrate that as the group number increases, the power to detect the association between $X$ and $Y$ also increases. However, in cases where too many groups are used, there will be insufficient power to estimate individual group estimates with sufficient precision. Group selection is therefore a trade-off between having a sufficient number of groups to power the analysis, and sufficient statistical power to estimate associations within each subgroup.

Whilst MRGxE may appear most suited to settings for which we have a categorical interaction covariate, this is not necessarily the case. In many situations, categorical variables simply use predefined groups, whilst implicitly measuring a continuous covariate. For example, frequency of alcohol consumption focuses upon quantity of alcohol consumed over time, which is essentially continuous. We therefore suggest researchers to be critical of subgrouping imposed by using categorical variables where continuous data are available.

As can be seen from Figure A2 it is also possible to infer the functional form of the interaction from the distribution of subgroups. The ordering of the subgroups can highlight the direction of the change in instrument-exposure association, whilst an observed bunching point can be indicative of inflection points. This is particularly clear where there is symmetry in the distribution of groups around such points.


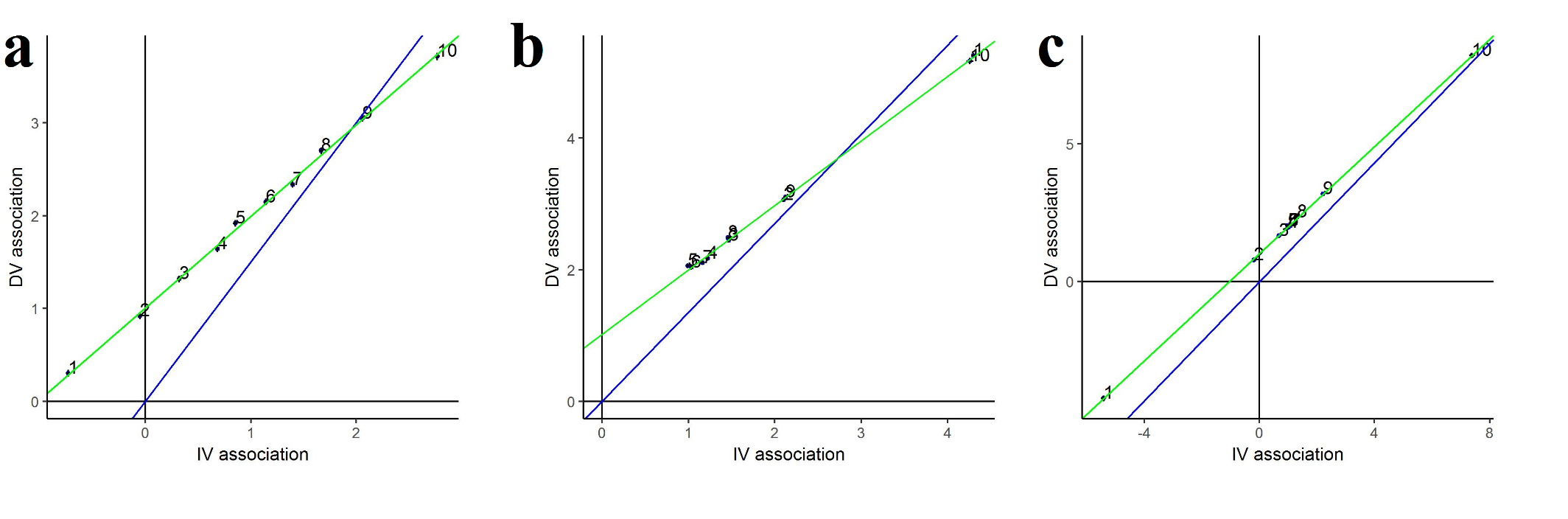


***Figure A2***: Three scatter plots showing the distribution of groups for the linear (a), quadratic (b), and cubic (c) interactions respectively. In each case, subgroups of $Z$ have been labelled in ascending order. For the linear interaction, the groups remain in ascending order of $Z$. In the quadratic case, subgroups show evidence of pairing symmetrical around the central fifth group. The distance between the extreme values of $Z$ is further indicative of the increasing difference in instrument-exposure association relative to the other groups. Finally, the cubic case shows a symmetrical cluster of groups around the fifth group, with extreme values of $Z$ lying further from the remaining groups.

# R code for implementing MRGXE using 5 interaction-covariate groups and a continuous interaction covariate.

Let G, Z, X, and Y be your instrument, interaction-covariate, exposure, and outcome respectively.

#Construct data frame containing G,Z,X, and Y

Data<-data.frame(Y,X,G,Z)

#Divide the sample into 5 equal interaction-covariate subgroups

Z.Bins <- quantile(Z,seq(0.2,1,0.2))

SubZ1 <- Data[which(Z < Z.Bins[1]),]

SubZ2 <- Data[which(Z < Z.Bins[2] & Z1 > Z.Bins[1]),]

SubZ3 <- Data[which(Z < Z.Bins[3] & Z1 > Z.Bins[2]),]

SubZ4 <- Data[which(Z < Z.Bins[4] & Z1 > Z.Bins[3]),]

SubZ5 <- Data[which(Z > Z.Bins[4]),]

#Estimate the G-X association, standard error, and mean F statistic for each interaction-covariate subgroup

GX_Function <- function(DAT){

FSR.Gen <- lm(X~G,data=DAT)

Std.Error <- summary(FSR.Gen)$coefficients[2,2]

return(list(FSR.Gen$coefficients[2],Std.Error,summary(FSR.Gen)$fstatistic[1]))

}

Gamma1 <- mapply(GX_Function,list(SubZ1,SubZ2,SubZ3,SubZ4,SubZ5))

Gamma1Ef <- unlist(Gamma1[1,])

Gamma1Err <- unlist(Gamma1[2,])

F.Stats <- unlist(Gamma1[3,])

#Estimate the G-Y association and standard error for each interaction-#covariate subgroup

GY_Function <- function(DAT){

RF.Gen <- lm(Y~G,data=DAT)

Std.Error <- summary(RF.Gen)$coefficients[2,2]

return(list(RF.Gen$coefficients[2],Std.Error))

}

Delta1 <- mapply(GY_Function,list(SubZ1,SubZ2,SubZ3,SubZ4,SubZ5))

Delta1Ef <- unlist(Delta1[1,])

Delta1Err <- unlist(Delta1[2,])

#Perform regression of G-Y associations upon G-X associations. The #intercept represents an estimate of #pleiotropic effect, whilst the slope #gives a corrected causal effect estimate.

MRGXE <- summary(lm(Delta1Ef~Gamma1Ef))

MRGXE

# Simulation demonstrating attenuation bias from transforming instrument-exposure associations to be positive

We return to the previous simulated example illustrating the effect of directional pleiotropy on estimates using OLS and TSLS regression (see Table A3). We introduce a further covariate$Z_{i}$, and divide the sample into 5 ascending groups of equal sizes, with group 1 exhibiting negative associations for both the instrument-exposure and instrument-outcome. The results for transforming the instrument-exposure association estimates under differing levels of induced pleiotropy are presented in Table A3 and Figure A3.

***Table A3****:* Comparison of transformation bias in IVW and MRGXE estimates under differing levels of pleiotropy

| **Induced Pleiotropy** | **Transformation** | **Model** | **Estimate**  **(true value=1)** | **95% CI** |
| --- | --- | --- | --- | --- |
| $\boldsymbol{\beta}_{\boldsymbol{2}}\boldsymbol{=0}$ | ***No*** | IVW  MRGXE (Intercept)  MRGXE (Effect) | 0.990  -0.014  0.998 | (0.96, 1.02)  (-0.10, 0.07)  (0.93, 1.06) |
|  | ***Yes*** | IVW  MRGXE (Intercept)  MRGXE (Effect) | 0.990  -0.058  1.027 | (0.96, 1.02)  (-0.11, 0.00)  (0.99, 1.07) |
| $\boldsymbol{\beta}_{\boldsymbol{2}}\boldsymbol{=1}$ | ***No*** | IVW  MRGXE (Intercept)  MRGXE (Effect) | 1.523  0.986  0.998 | (0.84, 2.21)  (0.90, 1.07)  (0.93, 1.06) |
|  | ***Yes*** | IVW  MRGXE (Intercept)  MRGXE (Effect) | 1.523  -0.148  1.616 | (0.84, 2.21)  (-2.53, 2.24)  (-0.13, 3.36) |

***Figure A3****:* Plots demonstrating bias introduced through effect transformation


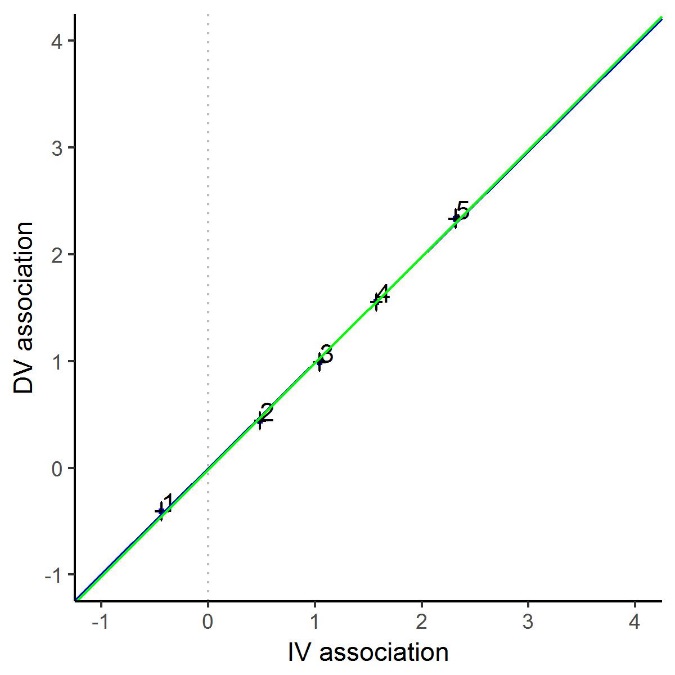

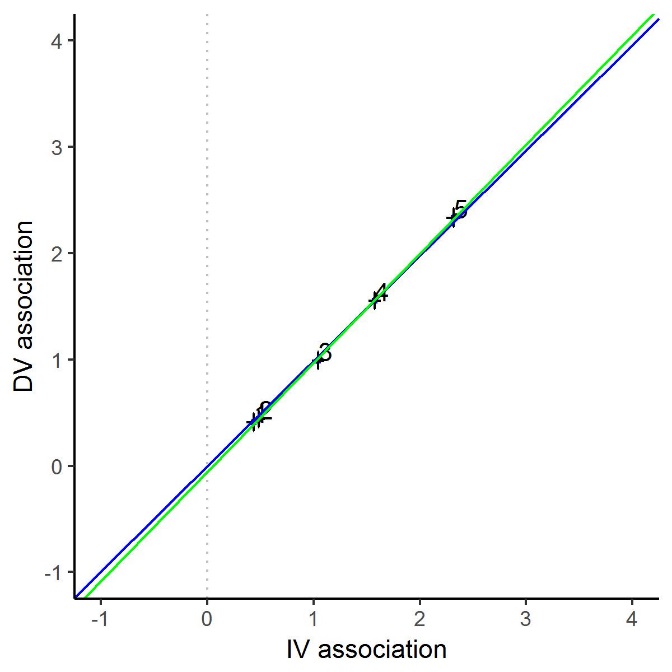


a) $\boldsymbol{\beta}_{\mathbf{2}}\mathbf{=0}$**, No transformation** b) $\boldsymbol{\beta}_{\mathbf{2}}\mathbf{=0}$**, Transformation**


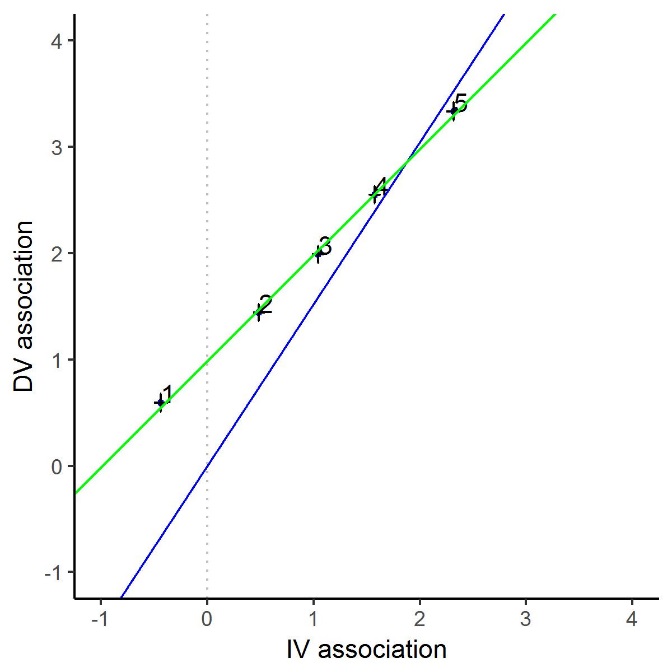

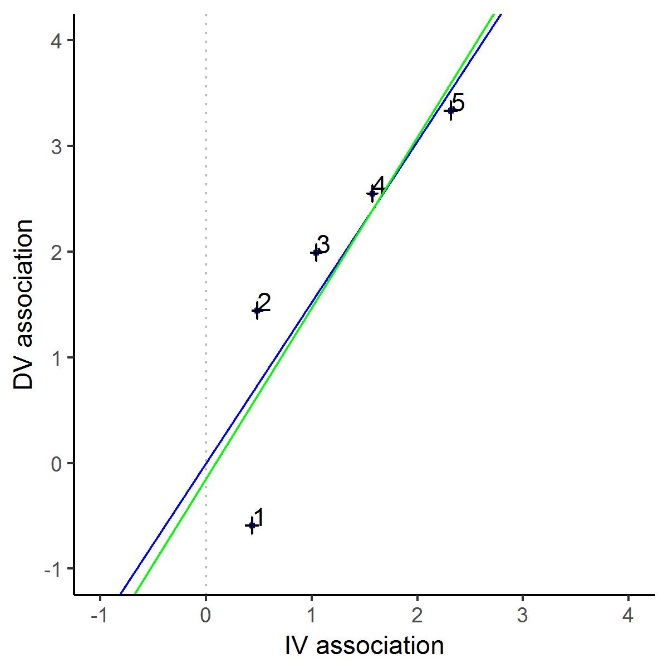


c) $\boldsymbol{\beta}_{\mathbf{2}}\mathbf{=1}$**, No Transformation** d) $\boldsymbol{\beta}_{\mathbf{2}}\mathbf{=1}$**, Transformation**

From Table A3 it can be seen that as expected the IVW estimate remains in agreement between transformed and non-transformed examples. In the balanced pleiotropy case ($\beta_{2}=0$), the MRGXE estimates remain accurate, whilst the effect estimates exhibit bias in the presence of directional pleiotropy ($\beta_{2}=1$). This is because transforming group 1 so as to have a positive instrument-exposure association has an impact upon the intercept estimate, as it is interpreted as group 1 having a similar instrument-exposure association to group 2. As a result, the intercept is underestimated, and subsequently the causal effect estimate is biased towards the IVW estimate.

# Simulation demonstrating relationship between pleiotropy estimate precision and distance of extrapolation

We perform a simulation using different mean values for the interaction covariate, an average directional pleiotropic effect of 1, and an exposure effect of 1. The results are shown in Figure A4 and Table A4, with greater precision in the pleiotropy estimate in scenario a) in comparison to scenario b). This can be viewed as a reflection of the instrument-covariate interaction strength in the first stage regression model, with stronger interactions contributing to more accurate effect estimates for $\beta_{1}$ and $\beta_{2}$.

***Figure A4****:* Plots demonstrating loss of precision due to increase in line extrapolation


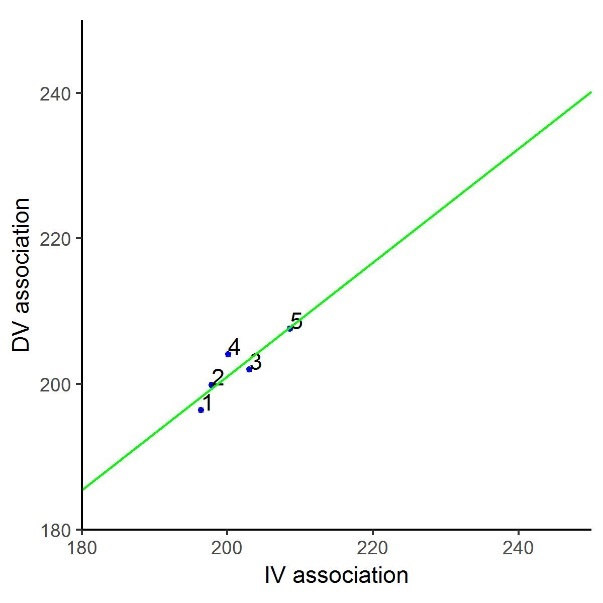

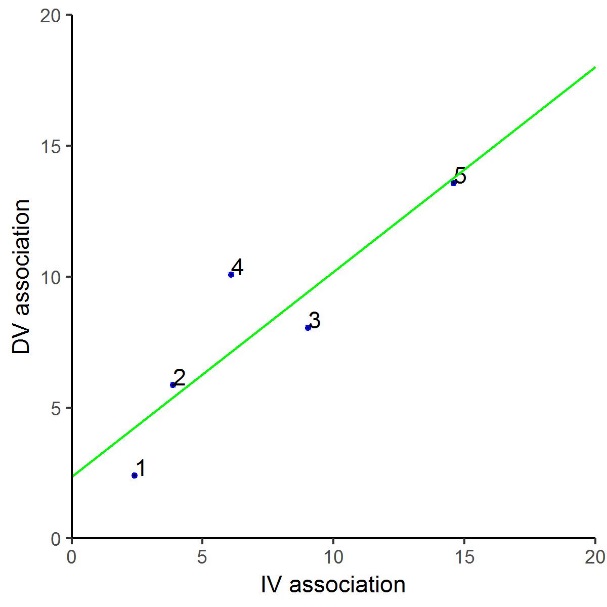


**a)** $\bar{\boldsymbol{Z}}\mathbf{=100, Var=2}$ **b)** $\bar{\boldsymbol{Z}}\mathbf{=3, Var=2}$

***Table A4***: Summary estimates for Figure A4 models

|  | Estimate | SE | 95% CI | p.value |
| --- | --- | --- | --- | --- |
| **Scenario a** |  |  |  |  |
| Intercept | 1.321 | 0.289 | (0.40, 2.24) | 0.020 |
| Effect$(\hat{\beta_{1}})$ | 0.997 | 0.003 | (0.99, 1.01) | <0.001 |
|  |  |  |  |  |
| **Scenario b** |  |  |  |  |
| Intercept | 1.010 | 0.013 | (0.97, 1.05) | <0.001 |
| Effect$(\hat{\beta_{1}})$ | 0.997 | 0.003 | (0.99, 1.01) | <0.001 |

In this example, the variance of the instrument-exposure associations has remained fixed, whilst the distance of the minimum $Z_{j}$ instrument-exposure association has been varied. Table A4 shows the comparable loss of precision in the pleiotropic effect estimate when increasing the distance of extrapolation, suggesting that the precision of the pleiotropy estimate is inversely proportional to the minimum distance of the $Z_{j}$ association from the y-axis. Note that increasing the variance of the instrument-exposure associations will result in an increase in precision as a consequence of utilising ordinary least squares regression.

# Derivation of the bias induced through violation of the constant pleiotropy assumption ($\boldsymbol{\beta}_{\boldsymbol{4}}\neq\boldsymbol{0}$).

To begin, define the following axioms using covariance and variance identities.

Axiom 1: For a constant $c$ and two random variables $H$ and $I$, the covariance of $cov\left( H,I+c \right)=cov\left( H,I \right)$

Axiom 2: For three random variables $H,I,J$, $cov\left( H+I,J \right)=cov\left( H,J \right)+cov\left( I,J \right)$

Axiom 3: For two random variables $H$ and $I$, $cov\left( 2H,I \right)=2cov\left( H,I \right)$

Axiom 4: For a random variable $H$, $cov\left( H,H \right)=var\left( H \right)$

Axiom 5: For a random variable $H$ and constant $a$, $var\left( aH \right)=a^{2}var\left( H \right)$

Let $G,Z,X,$ and $Y$ represent random variables and assume the following structural linear model:

$X_{i}=\gamma_{0}+\gamma_{1}G_{i}+\gamma_{2}Z_{i}+\gamma_{3}{G_{i}Z}_{i}+U_{i}+\varepsilon_{Xi}$ (A6)

$Y_{i}=\beta_{0}+\beta_{1}X_{i}+\beta_{2}G_{i}+\beta_{3}Z_{i}+\beta_{4}G_{i}Z_{i}+U_{i}+\varepsilon_{Yi}$ (A7)

We can construct a reduced form model by substituting (A6) into (A7):

$Y_{i}=\beta_{0}+\beta_{1}\left[ \gamma_{0}+\gamma_{1}G_{i}+\gamma_{2}Z_{i}+\gamma_{3}{G_{i}Z}_{i}+U_{i} \right]+\beta_{2}G_{i}+\beta_{3}Z_{i}+\beta_{4}G_{i}Z_{i}+U_{i}+({\varepsilon_{Xi}+\varepsilon}_{Yi})$ (A8)

It is then possible to estimate the partial effect of $G$ by taking the derivative of $G$ with respect to $X$ using (A6), and the derivative of $G$ with respect to $Y$ using (A8):

$\frac{dX}{dG}=\gamma_{1}+\gamma_{3}Z_{i}$ (A9)

$\frac{dY}{dG}=\beta_{1}\left( \gamma_{1}+\gamma_{3}Z_{i} \right)+\beta_{2}+\beta_{4}Z_{i}$ (A10)

Define the Wald estimand using (A9) and (A10) as:

$\frac{\beta_{1}\left( \gamma_{1}+\gamma_{3}Z_{i} \right)+\beta_{2}+\beta_{4}Z_{i}}{\gamma_{1}+\gamma_{3}Z_{i}}$ (A11)

The ordinary least squares (OLS) regression estimate can be obtained as:

$\frac{cov\left( \gamma_{1}+\gamma_{3}Z_{j}, \beta_{1}\left( \gamma_{1}+\gamma_{3}Z_{j} \right)+\beta_{2}+\beta_{4}Z_{j} \right)}{var\left( \gamma_{1}+\gamma_{3}Z_{j} \right)}$ (A12)

Where the subscript $j$ represents the $jth$interaction covariate subgroup.

From Axiom 1, $\beta_{2}$ can be removed from (A12):

$\frac{cov\left( \gamma_{1}+\gamma_{3}Z_{j}, \beta_{1}\left( \gamma_{1}+\gamma_{3}Z_{j} \right)+\beta_{4}Z_{j} \right)}{var\left( \gamma_{1}+\gamma_{3}Z_{j} \right)}$ (A13)

From Axiom 2:

$\frac{cov\left( \gamma_{1}+\gamma_{3}Z_{j}, \beta_{1}\left( \gamma_{1}+\gamma_{3}Z_{j} \right) \right)+cov\left( \gamma_{1}+\gamma_{3}Z_{j}, \beta_{4}Z_{j} \right)}{var\left( \gamma_{1}+\gamma_{3}Z_{j} \right)}$ (A14)

From Axiom 3:

$\frac{\beta_{1}cov\left( \gamma_{1}+\gamma_{3}Z_{j}, \gamma_{1}+\gamma_{3}Z_{j} \right)+\beta_{4}cov\left( \gamma_{1}+\gamma_{3}Z_{j}, Z_{j} \right)}{var\left( \gamma_{1}+\gamma_{3}Z_{j} \right)}$ (A15)

From Axiom 4:

$\frac{\beta_{1}var\left( \gamma_{1}+\gamma_{3}Z_{j} \right)+\beta_{4}cov\left( \gamma_{1}+\gamma_{3}Z_{j}, Z_{j} \right)}{var\left( \gamma_{1}+\gamma_{3}Z_{j} \right)}$ (A16)

From Axiom 1:

$\frac{\beta_{1}var\left( \gamma_{3}Z_{j} \right)+\beta_{4}cov\left( \gamma_{3}Z_{j}, Z_{j} \right)}{var\left( \gamma_{3}Z_{j} \right)}$ (A17)

From Axiom 3:

$\frac{\beta_{1}var\left( \gamma_{3}Z_{j} \right)+{\gamma_{3}\beta}_{4}cov\left( Z_{j}, Z_{j} \right)}{var\left( \gamma_{3}Z_{j} \right)}$ (A18)

From Axiom 4:

$\frac{\beta_{1}var\left( \gamma_{3}Z_{j} \right)+{\gamma_{3}\beta}_{4}var\left( Z_{j} \right)}{var\left( \gamma_{3}Z_{j} \right)}$ (A19)

From Axiom 5:

$$\frac{{\gamma_{3}^{2}\beta}_{1}var\left( Z_{j} \right)+{\gamma_{3}\beta}_{4}var\left( Z_{j} \right)}{\gamma_{3}^{2}var(Z_{j})}$$

$$=\frac{{\gamma_{3}^{2}\beta}_{1}+{\gamma_{3}\beta}_{4}}{\gamma_{3}^{2}}$$

$$=\frac{{\gamma_{3}\beta}_{1}+\beta_{4}}{\gamma_{3}}$$

$$=\beta_{1}+\frac{\beta_{4}}{\gamma_{3}}$$

(A20)

$$∎$$

# Derivation of the violations of the constant pleiotropy assumption through differing confounding structures.

**Scenario 1**


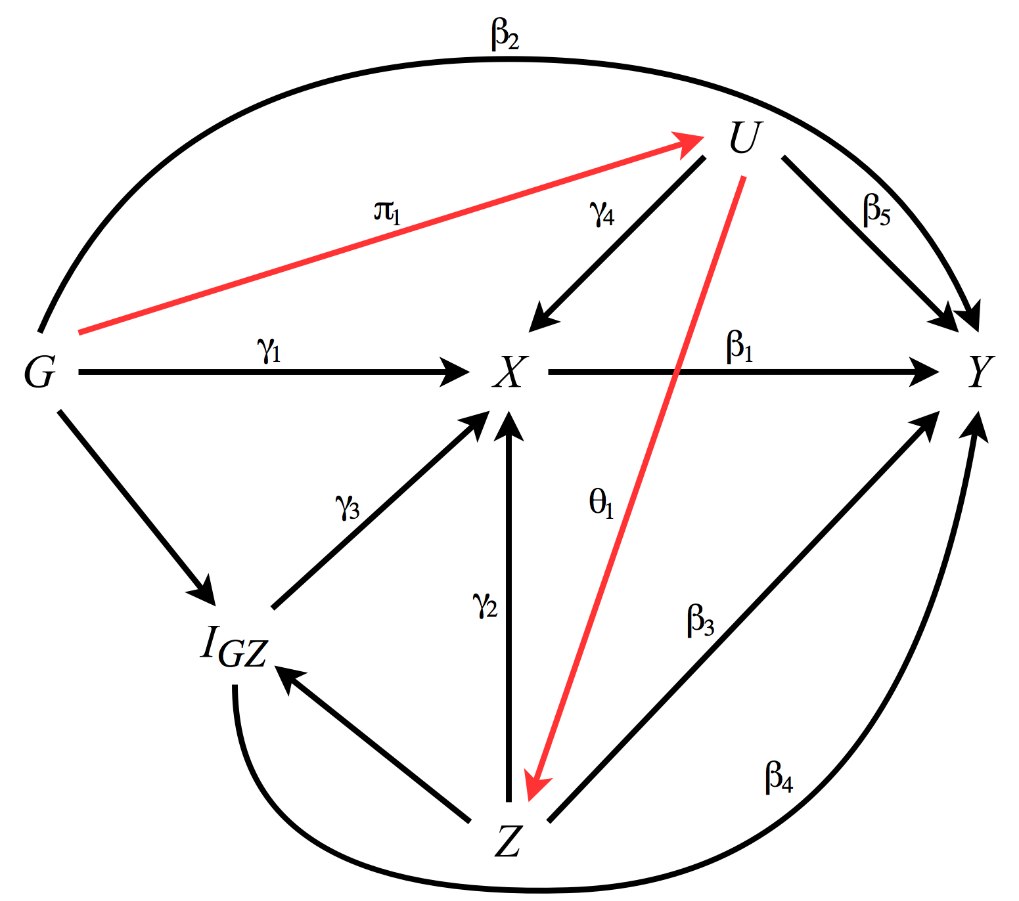


**Figure A5:** DAG illustrating a case in which the instrument $G$ is a determinant of the interaction-covariate $Z$ through a confounder $U$.

Data generating model

$G_{i}=N(0,1)$ (A21-1)

$U_{i}=\pi_{0}+\pi_{1}G_{i}+\epsilon_{U}$ (A21-2)

$Z_{i}=\theta_{0}+\theta_{1}U_{i}+\epsilon_{Z}$ (A21-3)

$X_{i}= \gamma_{0}+\gamma_{1}G_{i}+\gamma_{2}Z_{i}+\gamma_{3}GZ_{i}+\gamma_{4}U+\epsilon_{X}$ (A21-4)

$Y_{i}= \beta_{0}+\beta_{1}X_{i}+\beta_{2}G_{i}+\beta_{3}Z_{i}+\beta_{4}GZ_{i}+\beta_{5}U_{i}+\epsilon_{Y}$ (A21-5)

Definition of bias term

First a model for $X$ in terms of $G$ is constructed by substituting models (A21-2) and (A21-3) into model (A21-4):

$X_{i}= G_{i}\left( \gamma_{1}+\gamma_{2}\theta_{1}\pi_{1}+\gamma_{3}\theta_{1}\pi_{1}G_{i}+\gamma_{4}\pi_{1} \right)+\eta_{X}$ (A21-6)

We can also construct a model for the outcome $Y$ in terms of $G$ by substituting models (A21-2), (A21-3), and (A21-6) into model (A21-5):

$Y_{i}= G_{i}\left( \beta_{1}\left( \gamma_{1}+\gamma_{2}\theta_{1}\pi_{1}+\gamma_{3}\theta_{1}\pi_{1}G_{i}+\gamma_{4}\pi_{1} \right)+\beta_{2}+\beta_{3}\theta_{1}\pi_{1}+\beta_{4}\theta_{1}\pi_{1}G_{i}+\beta_{4}\pi_{4} \right)+\eta_{Y}$ (A21-7)

Using models (A21-6) and (A21-7), we can calculate the partial effect of $G$ for the first and second stage models respectively:

$\frac{dX}{dG}=\gamma_{1}+\gamma_{2}\theta_{1}\pi_{1}+2\gamma_{3}\theta_{1}\pi_{1}G_{i}+\gamma_{4}\pi_{1}$ (A21-8)

$\frac{dY}{dG}=\beta_{1}\left( \gamma_{1}+\gamma_{2}\theta_{1}\pi_{1}+2\gamma_{3}\theta_{1}\pi_{1}G_{i}+\gamma_{4}\pi_{1} \right)+\beta_{2}+\beta_{3}\theta_{1}\pi_{1}+2\beta_{4}\theta_{1}\pi_{1}G_{i}+\beta_{5}\pi_{1}$ (A21-9)

The corresponding Wald estimand is then given as:

$\beta_{1}+\frac{\beta_{2}+\beta_{3}\theta_{1}\pi_{1}+2\beta_{4}\theta_{1}\pi_{1}G_{i}+\beta_{5}\pi_{1}}{\gamma_{1}+\gamma_{2}\theta_{1}\pi_{1}+2\gamma_{3}\theta_{1}\pi_{1}G_{i}+\gamma_{4}\pi_{1}}$ (A21-10)

In equation (A21-10), the change in pleiotropic effect across the set of subgroups is represented by the term $2\beta_{4}\theta_{1}\pi_{1}G_{i}$. In this case, $\beta_{4}$ represents the average change in pleiotropic effect across subgroups, $\theta_{1}$ is the effect of $G$ upon the confounder $U$, and $\pi_{1}$ is the effect of the $G$ mediated by the confounder $U.$ This highlights three important elements of the constant pleiotropy assumption:

1. MRGXE will give an unbiased effect estimate $\beta_{1}$ in cases where there is no average change in pleiotropic effect across the set of subgroups $\left( \beta_{4}=0 \right)$. Importantly, this includes the possibility of changes in pleiotropic effect being balanced across the sample, in a similar fashion to the balanced pleiotropic effects in the MR Egger framework.
2. MRGXE will give an unbiased effect estimate $\beta_{1}$ when there is no effect of the instrument $G$ upon a confounder $\left( \theta_{1}=0 \right)$.
3. MRGXE will give an unbiased effect estimate $\beta_{1}$ when there is no effect of the instrument $G$ mediated by the confounder $\left( \pi_{1}=0 \right)$.

**Scenario 2:**


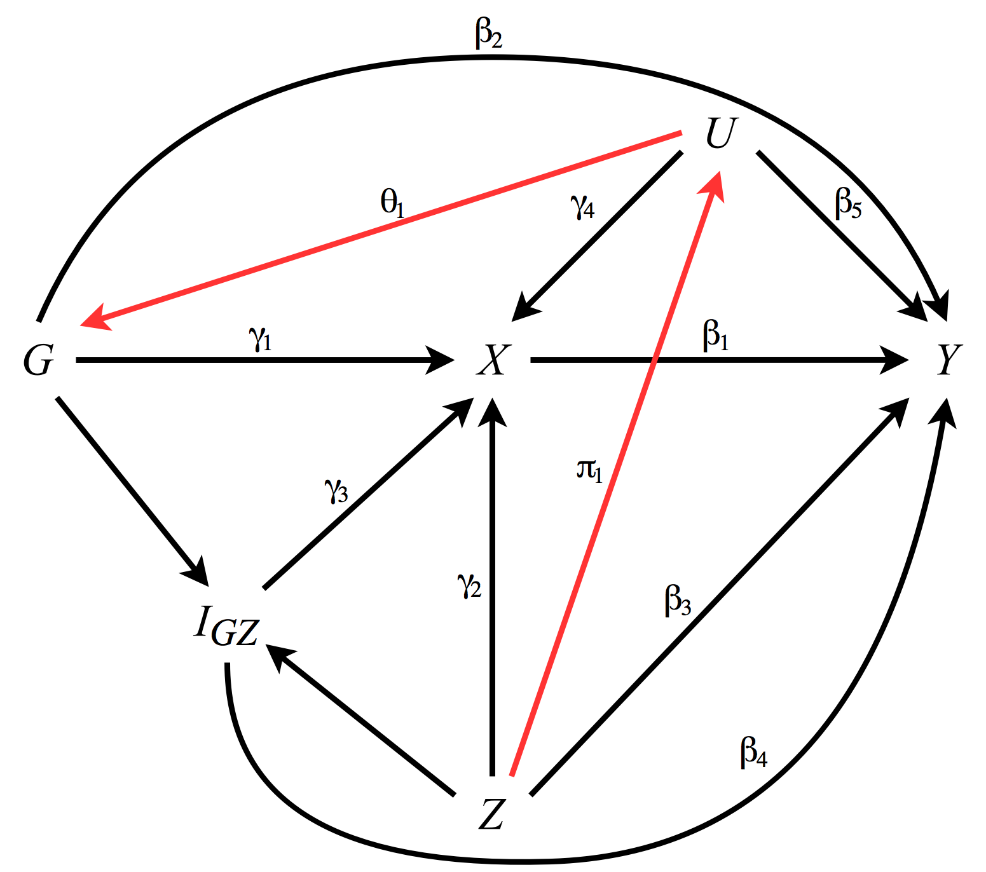


**Figure A6:** DAG illustrating a case in which the interaction-covariate $Z$ is a determinant of the instrument $G$ through a confounder $U$.

Data generating model

$Z_{i}=N(0,1)$ (A22-1)

$U_{i}=\pi_{0}+\pi_{1}Z_{i}+\epsilon_{U}$ (A22-2)

$G_{i}=\theta_{0}+\theta_{1}U_{i}+\epsilon_{G}$ (A22-3)

$X_{i}= \gamma_{0}+\gamma_{1}G_{i}+\gamma_{2}Z_{i}+\gamma_{3}GZ_{i}+\gamma_{4}U+\epsilon_{X}$ (A22-4)

$Y_{i}= \beta_{0}+\beta_{1}X_{i}+\beta_{2}G_{i}+\beta_{3}Z_{i}+\beta_{4}GZ_{i}+\beta_{5}U_{i}+\epsilon_{Y}$ (A22-5)

Definition of bias term

First model for the exposure $X$ in terms of $Z$ is constructed by substituting models (A22-2) and (A22-3) into model (A22-4):

$X_{i}= Z_{i}\left( \gamma_{1}\theta_{1}\pi_{1}+\gamma_{2}+\gamma_{3}\theta_{1}\pi_{1}Z_{i}+\gamma_{4}\pi_{1} \right)+\eta_{X}$ (A22-6)

We can also construct a model for the outcome $Y$ in terms of $Z_{i}$ by substituting models (A22-2), (A22-3), and (A22-6) into model (A22-5):

$Y_{i}= Z_{i}\left( \beta_{1}\left( \gamma_{1}\theta_{1}\pi_{1}+\gamma_{2}+\gamma_{3}\theta_{1}\pi_{1}Z_{i}+\gamma_{4}\pi_{1} \right)+\beta_{2}\theta_{1}\pi_{1}+\beta_{3}+\beta_{4}\theta_{1}\pi_{1}Z_{i}+\beta_{5}\pi_{1} \right)+\eta_{Y}$ (A22-7)

As $Z$ is a determinant of both $U$ and $G$, it is appropriate to calculate the partial effect of $Z$ with respect to $X$ and $Y$

$\frac{dX}{dZ}=\gamma_{1}\theta_{1}\pi_{1}+\gamma_{2}+{2\gamma}_{3}\theta_{1}\pi_{1}Z_{i}+\gamma_{4}\pi_{1}$ (A22-8)

$\frac{dY}{dZ}=\beta_{1}\left( \gamma_{1}\theta_{1}\pi_{1}+\gamma_{2}+{2\gamma}_{3}\theta_{1}\pi_{1}Z_{i}+\gamma_{4}\pi_{1} \right)+\beta_{2}\theta_{1}\pi_{1}+\beta_{3}+2\beta_{4}\theta_{1}\pi_{1}Z_{i}+\beta_{5}\pi_{1}$ (A22-9)

The corresponding Wald estimand is then given as:

$\beta_{1}+\frac{\beta_{2}\theta_{1}\pi_{1}+\beta_{3}+2\beta_{4}\theta_{1}\pi_{1}Z_{i}+\beta_{5}\pi_{1}}{\gamma_{1}\theta_{1}\pi_{1}+\gamma_{2}+{2\gamma}_{3}\theta_{1}\pi_{1}Z_{i}+\gamma_{4}\pi_{1}}$ (A22-10)

In this case term $2\beta_{4}\theta_{1}\pi_{1}Z_{i}$ has an equivalent role in invalidating MRGXE estimates as the $2\gamma_{3}\theta_{1}\pi_{1}G_{i}$ term from scenario 1.

**Scenario 3:**


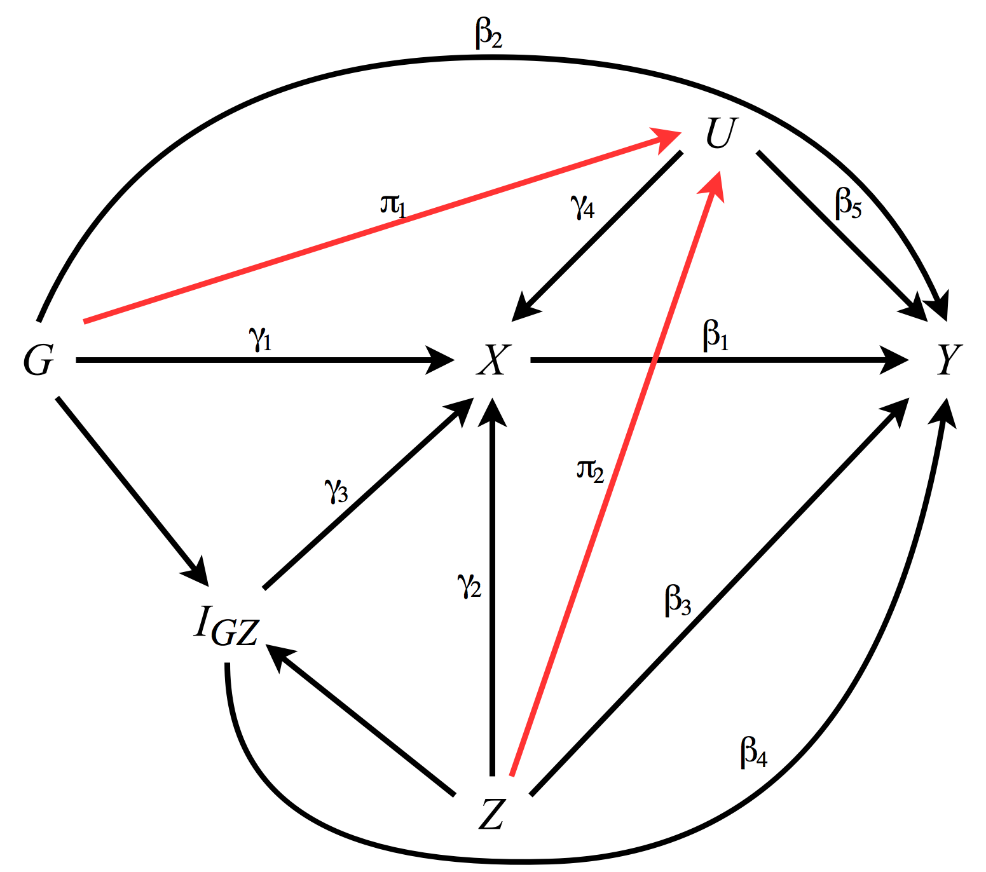


**Figure A7:** DAG illustrating a case in which the interaction-covariate $Z$ and the instrument $G$ are determinants of the confounder $U$.

Data generating model

$G_{i}=N(0,1)$ (A23-1)

$Z_{i}=N(0,1)$ (A23-2)

$U_{i}=\pi_{0}+\pi_{1}G_{i}+\pi_{2}Z_{i}+\epsilon_{U}$ (A23-3)

$X_{i}= \gamma_{0}+\gamma_{1}G_{i}+\gamma_{2}Z_{i}+\gamma_{3}GZ_{i}+\gamma_{4}U+\epsilon_{X}$ (A23-4)

$Y_{i}= \beta_{0}+\beta_{1}X_{i}+\beta_{2}G_{i}+\beta_{3}Z_{i}+\beta_{4}GZ_{i}+\beta_{5}U_{i}+\epsilon_{Y}$ (A23-5)

Definition of bias term

First a model for the exposure $X$ in terms of $G$ is constructed by substituting models (A23-2) and (A23-3) into model (A23-4):

$X_{i}= G_{i}\left( \gamma_{1}+\gamma_{3}Z_{i}+\gamma_{4}\pi_{1} \right)+\eta_{X}$ (A23-6)

We can also construct a model for the outcome $Y$ in terms of $G$ by substituting models (A23-2), (A23-3), and (A23-6) into model (A23-5):

$Y_{i}= G_{i}\left( \beta_{1}\left( \gamma_{1}+\gamma_{3}Z_{i}+\gamma_{4}\pi_{1} \right)+\beta_{2}+\beta_{4}Z_{i}+\beta_{5}\pi_{1} \right)+\eta_{Y}$ (A23-7)

Using models (A23-6) and (A23-7), we can calculate the partial effect of $G$ for the first and second stage models respectively:

$\frac{dX}{dG}=\gamma_{1}+\gamma_{3}Z_{i}+\gamma_{4}\pi_{1}$ (A23-8)

$\frac{dY}{dG}=\beta_{1}\left( \gamma_{1}+\gamma_{3}Z_{i}+\gamma_{4}\pi_{1} \right)+\beta_{2}+\beta_{4}Z_{i}+\beta_{5}\pi_{1}$ (A23-9)

The corresponding Wald estimand is then given as:

$\beta_{1}+\frac{\beta_{2}+\beta_{4}Z_{i}+\beta_{5}\pi_{1}}{\gamma_{1}+\gamma_{3}Z_{i}+\gamma_{4}\pi_{1}}$ (A23-10)

As can be seen from equation (A23-10), the effect of $G$ through the confounder $U$ is constant and incorporated into the MRGXE pleiotropy correction. As a consequence, MRGXE provides an unbiased estimate of the causal effect $\beta_{1}$.

**Scenario 4:**


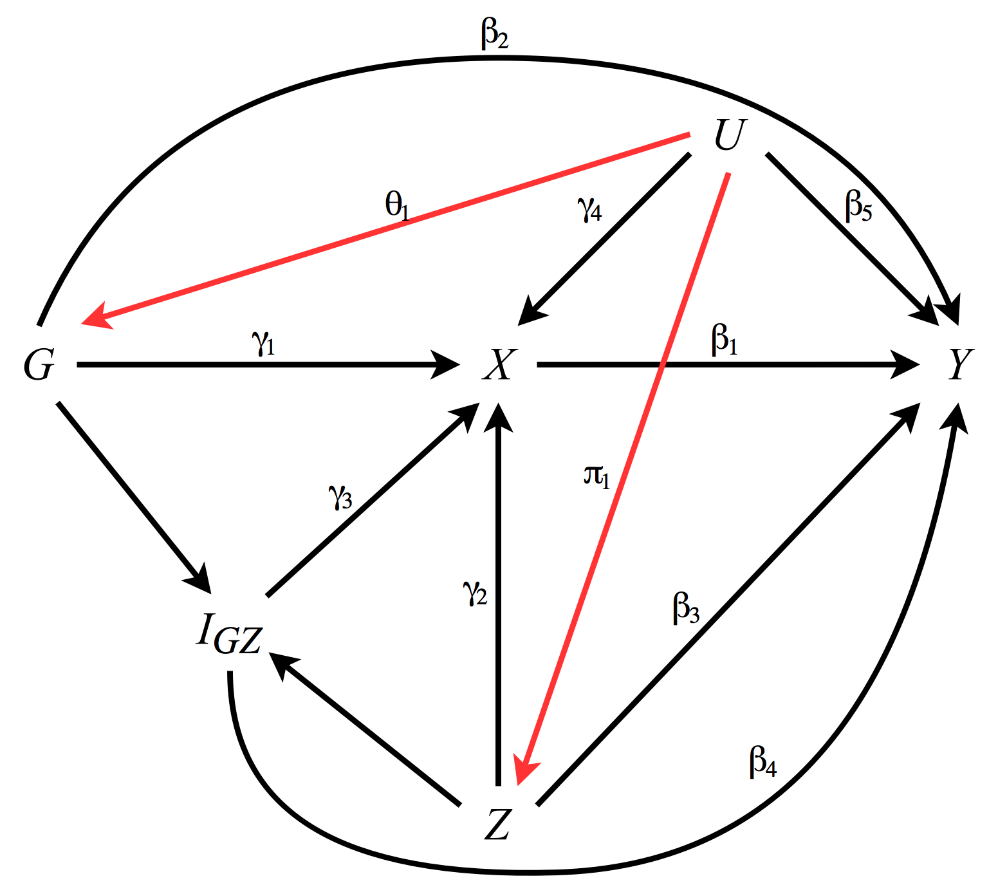


**Figure A8:** DAG illustrating a case in which the confounder $U$ is a determinant of both the interaction-covariate $Z$ and the instrument $G$.

Data generating model

$U_{i}=N(0,1)$ (A24-1)

$Z_{i}=\pi_{0}+\pi_{1}U_{i}+\epsilon_{Z}$ (A24-2)

$G_{i}=\theta_{0}+\theta_{1}U_{i}+\epsilon_{G}$ (A24-3)

$X_{i}= \gamma_{0}+\gamma_{1}G_{i}+\gamma_{2}Z_{i}+\gamma_{3}GZ_{i}+\gamma_{4}U+\epsilon_{X}$ (A24-4)

$Y_{i}= \beta_{0}+\beta_{1}X_{i}+\beta_{2}G_{i}+\beta_{3}Z_{i}+\beta_{4}GZ_{i}+\beta_{5}U_{i}+\epsilon_{Y}$ (A24-5)

Definition of bias term

We can substitute model (A24-2) into models (A24-4) and (A24-5), such that

$X_{i}= U_{i}\left( \gamma_{1}\theta_{1}+\gamma_{2}\pi_{1}+\gamma_{3}\theta_{1}{\pi_{1}U}_{i}+\gamma_{4} \right)+\eta_{X}$ (A24-6)

$$Y_{i}= U_{i}\left( \beta_{1}\left( \gamma_{1}\theta_{1}+\gamma_{2}\pi_{1}+2\gamma_{3}\theta_{1}\pi_{1}U_{i}+\gamma_{4} \right)+\beta_{2}\theta_{1}+\beta_{3}\pi_{1}+{2\beta}_{4}\theta_{1}\pi_{1}U_{i}+\beta_{5} \right)+\eta_{Y}$$

As the instrument $G$ is determined by $U$, it is appropriate to calculate the partial effect of $U$ for the first and second stage models respectively:

$\frac{dX}{dU}=\gamma_{1}\theta_{1}+\gamma_{2}\pi_{1}+2\gamma_{3}\theta_{1}\pi_{1}U_{i}+\gamma_{4}$ (A24-8)

$\frac{dY}{dU}=\beta_{1}\left( \gamma_{1}\theta_{1}+\gamma_{2}\pi_{1}+2\gamma_{3}\theta_{1}\pi_{1}U_{i}+\gamma_{4} \right)+\beta_{2}\theta_{1}+\beta_{3}\pi_{1}+{2\beta}_{4}\theta_{1}\pi_{1}U_{i}+\beta_{5}$ A24- (9)

The corresponding Wald estimand is then given as:

$\beta_{1}+\frac{\beta_{2}\theta_{1}+\beta_{3}\pi_{1}+{2\beta}_{4}\theta_{1}\pi_{1}U_{i}+\beta_{5}}{\gamma_{1}\theta_{1}+\gamma_{2}\pi_{1}+2\gamma_{3}\theta_{1}\pi_{1}U_{i}+\gamma_{4}}$ (A24-10)

As with scenario 1 and scenario 2, the term ${2\beta}_{4}\theta_{1}\pi_{1}U_{i}$ encapsulates the bias resulting from change in pleiotropic effects across subgroups. As a result, the MRGXE model will be invalidated where there are one or more confounders causally downstream of both the instrument $G$ and the interaction covariate $Z$, and where the average effect of the set of confounders is not balanced.

# Observational and TSLS estimates using the UK Biobank sample

***Table A5***: OLS and TSLS effect estimates using the UK Biobank sample. The OLS estimates are obtained by regressing SBP upon BMI and TDI, whilst the TSLS estimates utilise the weighted allelic score as an instrument for BMI.

|  | Estimate | SE | 95% CI | p.value |
| --- | --- | --- | --- | --- |
| **OLS** |  |  |  |  |
| Intercept | <0.001 | 0.002 | (-0.003, 0.003) | >0.999 |
| BMI | 0.194 | 0.002 | (0.19, 0.20) | <0.001 |
| TDI | -0.060 | 0.002 | (-0.063, -0.057) | <0.001 |
| **TSLS** |  |  |  |  |
| Intercept | <0.001 | 0.002 | (-0.003, 0.003) | >0.999 |
| BMI | 0.106 | 0.012 | (0.08, 0.13) | <0.001 |
| TDI | -0.053 | 0.002 | (-0.06, -0.05) | <0.001 |

# Scatter plots for differing group selection in MRGXE applied analysis


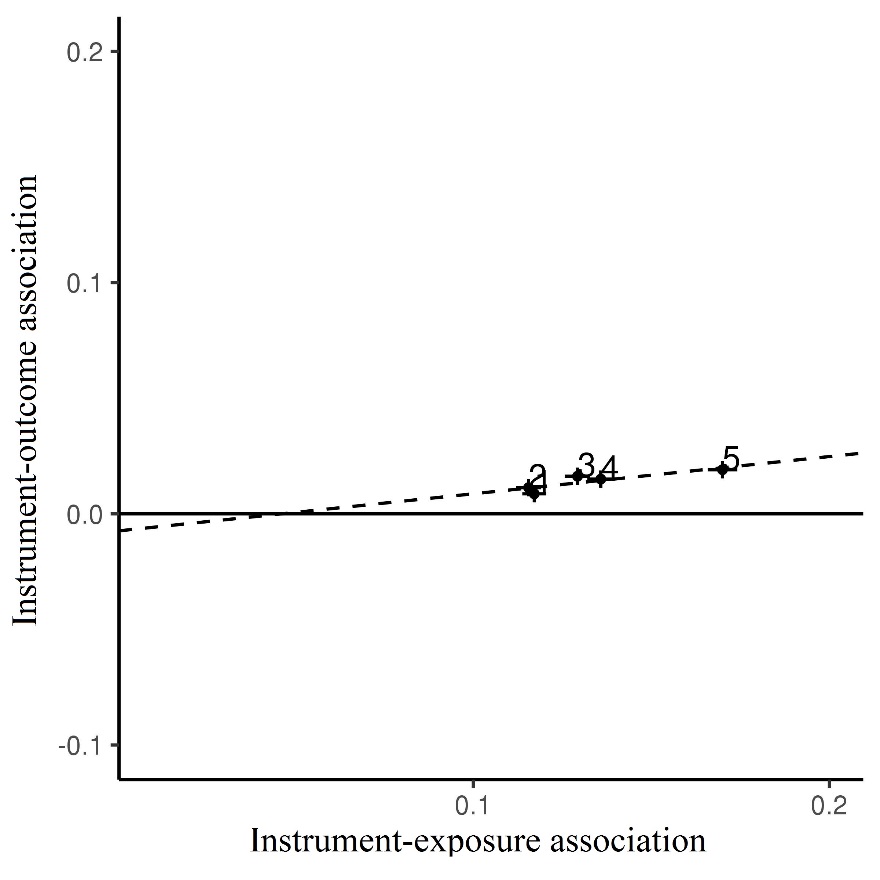


***Figure A9***: Plot showing MRGXE estimate using 5 group TDI subgroups


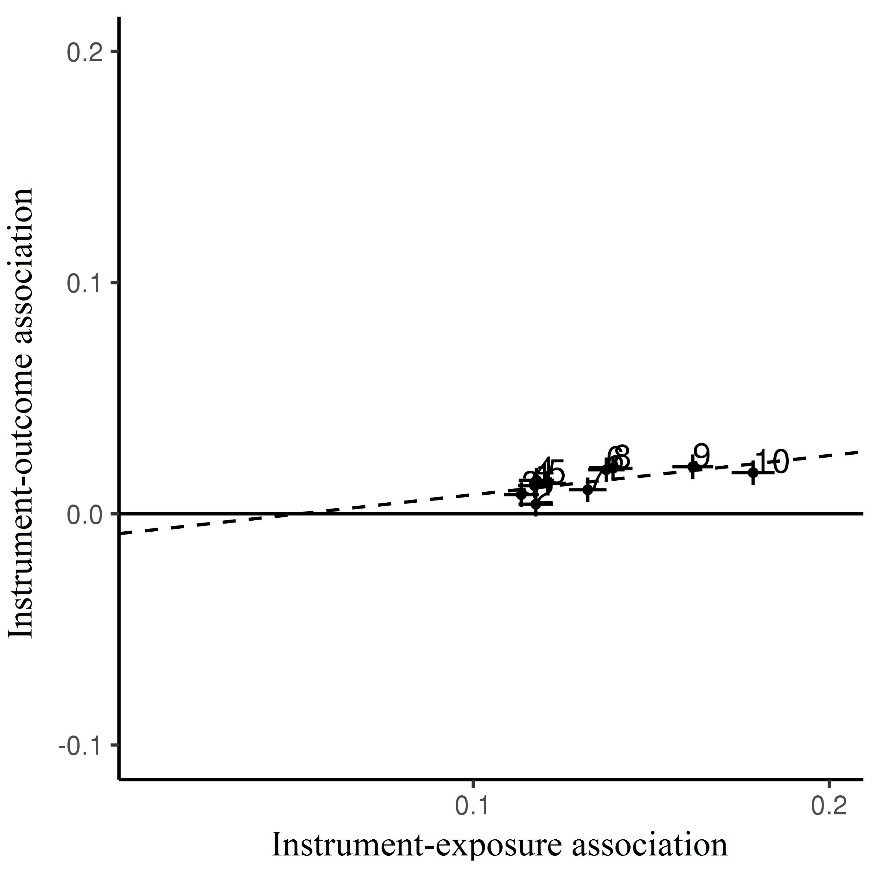


***Figure A10***: Plot showing MRGXE estimate using 10 group TDI subgroups


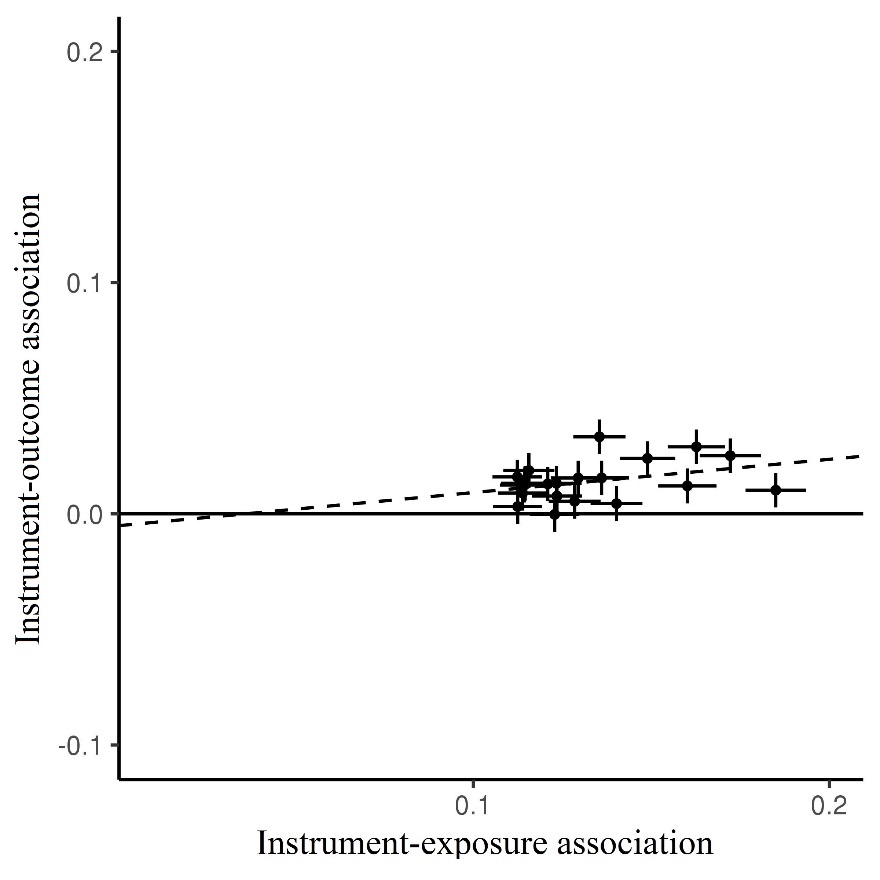


***Figure A11***: Plot showing MRGXE estimate using 20 group TDI subgroups (group numbers omitted for clarity)


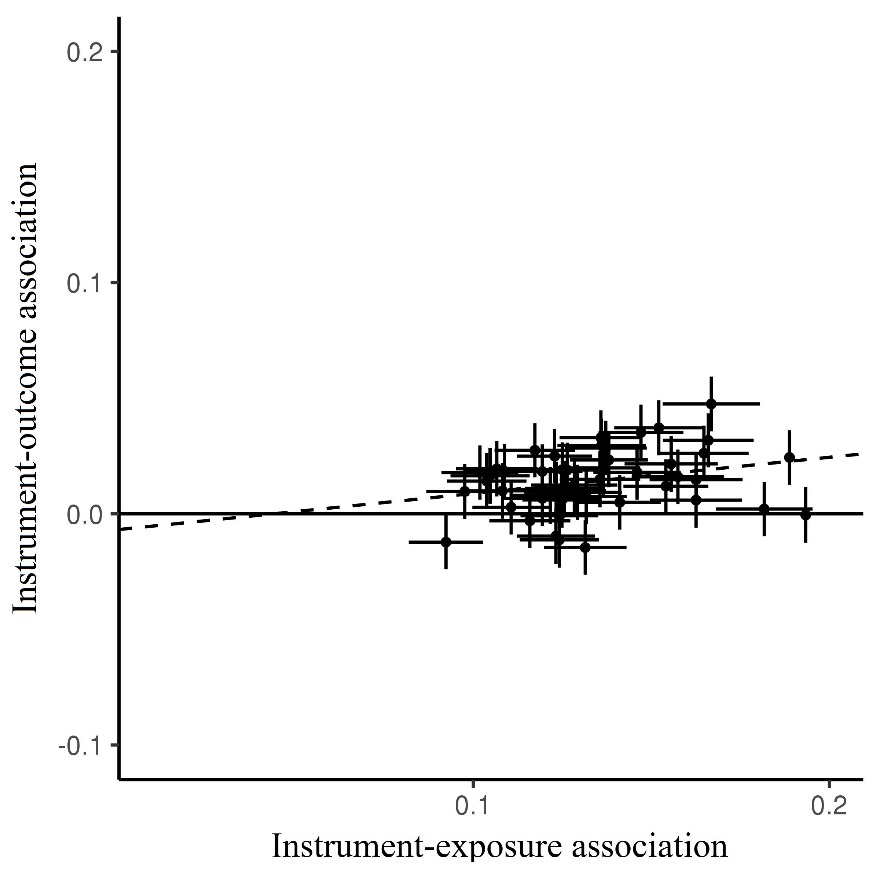


***Figure A12***: Plot showing MRGXE estimate using 50 group TDI subgroups (group numbers omitted for clarity)

# Simulation specifications

The simulation study was performed by initially generating the following random variables for a given sample size $N$:

$$G\sim B(1, 0.6)$$

$$Z\sim N(1, 0.6)$$

Confounding was induced by generating two vectors $\varepsilon_{X}$ and $\varepsilon_{Y}$ from a multivariate normal distribution, such that the correlation between $\varepsilon_{X}$ and $\varepsilon_{Y}$ is 0.5 (Pearson).

Values for $X$ and $Y$ were generated using the following linear models:

$X_{i}=1+\gamma_{1}G_{i}+\gamma_{2}Z_{i}+\gamma_{3}{GZ}_{i}+\varepsilon_{Xi}$ (A25)

$Y_{i}=1+\beta_{1}X_{i}+\beta_{2}G_{i}+\beta_{3}Z_{i}+\beta_{4}G_{i}Z_{i}+\varepsilon_{Yi}$ (A26)

The values of $\gamma_{1},$ $\gamma_{2}$, $\gamma_{3}$, and $\beta_{3}$ were fixed at 1, whilst $\beta_{1},\beta_{2}$, and $\beta_{4}$ vary as indicated in the paper.
